# Analysis of the anaerobic digestion metagenome under environmental stresses stimulating prophage induction

**DOI:** 10.1186/s40168-022-01316-w

**Published:** 2022-08-15

**Authors:** Alessandro Rossi, Maria Silvia Morlino, Maria Gaspari, Arianna Basile, Panagiotis Kougias, Laura Treu, Stefano Campanaro

**Affiliations:** 1 Department of Biology, University of Padua, via U. Bassi 58/b, 35131 Padova, Italy; 2 Department of Hydraulics, Soil Science and Agricultural Engineering, Faculty of Agriculture, Aristotle University of Thessaloniki, GR-54124 Thessaloniki, Greece; 3 Soil and Water Resources Institute, Hellenic Agricultural Organisation Demeter, Thermi, 57001 Thessaloniki, Greece; 4 CRIBI biotechnology center, University of Padua, via U. Bassi 58/b, 35131 Padova, Italy

## Abstract

**Background:**

The viral community has the potential to influence the structure of the microbiome and thus the yield of the anaerobic digestion process. However, the virome composition in anaerobic digestion is still under-investigated. A viral induction experiment was conducted on separate batches undergoing a series of DNA-damaging stresses, in order to coerce temperate viruses to enter the lytic cycle.

**Results:**

The sequencing of the metagenome revealed a viral community almost entirely composed of tailed bacteriophages of the order *Caudovirales*. Following a binning procedure 1,092 viral and 120 prokaryotic genomes were reconstructed, 64 of which included an integrated prophage in their sequence.

Clustering of coverage profiles revealed the presence of species, both viral and microbial, sharing similar reactions to shocks. A group of viral genomes, which increase under organic overload and decrease under basic pH, uniquely encode the *yopX* gene, which is involved in the induction of temperate prophages. Moreover, the in-silico functional analysis revealed an enrichment of sialidases in viral genomes. These genes are associated with tail proteins and, as such, are hypothesised to be involved in the interaction with the host. *Archaea* registered the most pronounced changes in relation to shocks and featured behaviours not shared with other species. Subsequently, data from 123 different samples of the global anaerobic digestion database was used to determine coverage profiles of host and viral genomes on a broader scale.

**Conclusions:**

Viruses are key components in anaerobic digestion environments, shaping the microbial guilds which drive the methanogenesis process. In turn, environmental conditions are pivotal in shaping the viral community and the rate of induction of temperate viruses. This study provides an initial insight into the complexity of the anaerobic digestion virome and its relation with the microbial community and the diverse environmental parameters.

Video Abstract

**Supplementary Information:**

The online version contains supplementary material available at 10.1186/s40168-022-01316-w.

## Background

Anaerobic digestion (AD) is a functional process carried out by microbial communities composed of *Bacteria* and *Archaea* which degrade organic matter in anoxic conditions. AD occurs in natural environments such as aquatic sediments, wetlands, and animal gut, but it is also widely employed in industrial processes. It is particularly valuable as a way to produce methane while disposing of organic waste, playing an important role in the reduction of the dependence from fossil fuels and the development of a circular economy approach [1].

The composition of AD microbiomes is extremely variable, and it reflects the wide variety of substrates and physicochemical conditions under which this degradation process occurs, both in natural and technical environments [2]. In AD, polymers are first broken down into simple molecules, which are then converted into Volatile Fatty Acids (VFA), then into acetate and finally into methane in the four steps of hydrolysis, acidogenesis, acetogenesis and methanogenesis. The first three steps are conducted by the bacterial community which, despite the great variation across different conditions, is dominated by the phylum *Firmicutes*, usually followed by *Bacteroidetes* and *Proteobacteria*. Archaeal species, mostly belonging to the phylum *Euryarchaeota*, are involved in the conversion of simple molecules to methane and usually account for a much smaller part of the community [3]. The microbial species present and their balance are crucial for optimisation of biogas production, and they have been extensively studied in the last two decades [4]. Among the numerous factors concurring to shape microbial communities, the importance of viruses, in particular bacteriophages, is increasingly recognised [5]. Viral concentration in samples from wastewater treatment plants (WWTPs) has been estimated to be greater in comparison to aquatic environments by one to three orders of magnitude [6, 7]. Furthermore, it has been observed that bacteriophages have a strong correlation with prokaryotic species across time in wastewater-treating bioreactors [8]. Despite the importance of this, most of the existing articles regarding the AD virome are limited to characterisations of the community and do not assess the impact of viruses on the microbial community [9–11]. Zhang and colleagues showed that there is a correlation between the viral community composition and the production of methane in anaerobic digesters of WWTPs and argued that the viral shunt has a positive impact on the production of methane [12], but such conclusions are drawn on a broad scale analysis, leaving many of the actual dynamics unaddressed.

The transition of a temperate phage from lysogenic to lytic cycle is known as induction. Temperate viruses spontaneously undergo induction at a low rate, but in several species of bacteriophages and archaeal viruses, this phenomenon is known to increase with DNA-damaging stresses [13]. For example, in a study targeting the response to different types of anaerobic stresses in *Nitrosospira multiformis* 25196, it was observed how *N. multiformis* cells reacted to a wide range of environmental stresses through prophage induction [14].

A prime example of the importance of bacteriophages in engineered systems is the dairy industry, which is threatened by bacteriophages attacking *Streptococcus thermophilus* strains [15]. Moreover, viruses are known to be players in the regulation of global carbon and nitrogen cycles in natural ecosystems [12], e.g. aquifer sediments [16], and in phytoplankton dynamics and diversity [17].

As parasites, viruses apply strong selective pressures on their hosts. It has been estimated that in marine ecosystems, viruses kill about 20% of the microbial biomass daily [18]. The recycling of organic matter from lysed microbes, called viral shunt, plays a relevant role in the regulation of global carbon and nitrogen cycles. In biogas plants, phage-induced bacterial cell lysis can decrease biogas production when the key species associated with biogas production are affected. At the same time, auxotrophic microorganisms are benefitted as lysis serves as a source of cofactors, vitamins and amino acids [11]. Furthermore, as mobile genetic elements, viruses enact horizontal gene transfer (HGT) across microbes at different taxonomic ranks, from species to phyla. This potentially endows hosts with beneficial functions, increases the genetic diversity of the population, and plays a role in the complex co-evolutionary dynamics between viruses and hosts [19]. However, both HGT and viral lysis rates in engineered systems are still overlooked.

Despite the elucidation of virus-mediated mechanisms, most of the viral diversity remains unknown [20]. However, the advent of metagenomics has brought large advances in the description of microbial environmental communities. The introduction of binning methods in standard metagenomic data analysis pipelines has allowed for the recovery of many uncultivable AD microbial species [2] and the detailed description of key organisms of the microbiome. The exponential increase of available sequences from both bulk metagenomes and metaviromes has led to the creation of numerous databases of viral sequences [21, 22], paralleled by the development of predictors able to effectively find new phages [23–26]. All viral prediction algorithms depend to some extent on the previous knowledge associated with taxonomically assigned genomes reported in public databases. This applies to all the software, whether they are based on homology search, like CheckV and PHASTER, or leverage k-mer usage like VirFinder, or analyse sequence features within machine learning frameworks, like VIBRANT, VirSorter2 and PPR-Meta [23–28]. However, their application has proven effective in discovering novel viral clades, the most emblematic case being the crAss-like phage family [29–31]. In the light of the relevant results obtained from metaviromics the International Committee on Taxonomy of Viruses proposed the establishment of new classification methods based solely on genomic features [32].

Unravelling the “dark matter” of novel viral diversity is a daunting task, and the aforementioned exploratory studies conducted on the AD virome showed the potential that phages have in shaping the prokaryotic community [9, 11, 12]. The DNA virome of AD has been described as dominated by tailed bacteriophages of the *Siphoviridae*, *Podoviridae* and *Myoviridae* families, with a minor presence of *Tectiviridae*, *Inoviridae* and other families. The AD microbiome is extremely complex and composed of species involved in different functional tasks, including the hydrolysis of organic matter and the conversion of the derived by-products in simple organic molecules (e.g. volatile fatty acids and methane). However, little is known on which prokaryotic species can be potentially affected by phages and, therefore, which are the functional processes potentially influenced by lytic cycles. Heyer and colleagues [11] reported that species belonging to *Bacillaceae*, *Enterobacteriaceae*, and *Clostridiaceae* are among the favourite targets of bacteriophages, but these findings are not conclusive to determine whether specific parts of the AD funnel are more impacted by viruses. Identifying and characterising the viruses and their hosts in this system can lead not only to a better comprehension of AD microbial dynamics but also to applications such as phage-mediated treatment of the reactors in order to increase process performance. Bacteriophages are already used as tools for manipulating microbial communities in different fields, such as phage therapy and pathogen control in food and water, and have been used as control for biomass bulking in wastewater treatment [33–36]. An increased attention towards the AD viral community could lead to the development of similar techniques for the improvement of the AD process as well. This could be achieved by removing species like the sulphate reducers which compete with key players in pivotal steps of methanogenesis, or leveraging bacteriophage-mediated HGT in order to confer desirable metabolic characteristics to microbial species of interest. In this experiment, we attempted to use induction to explore the effect of diverse conditions potentially affecting the AD process on both the microbial and viral community. We then assessed the presence of the retrieved genomes in other AD metagenomes from the Biogas Microbiome collection [2, 37] (microbial-genomes.org).

## Materials and methods

### Inoculum and feedstock

Active inoculum was obtained from a lab-scale Continuous Stirred Tank Reactor (CSTR) (Waste Management and Bioprocessing Lab, Thessaloniki, Greece), treating cattle manure at mesophilic conditions (37 ± 1 °C). Cattle manure was collected from a full-scale biogas plant located in northern Greece (Biogas Lagada S.A., Thessaloniki, Greece). The raw substrate was sieved using a separating net with a 2 mm opening to remove large particles and stored until usage at −20 °C to prevent alterations in its composition.

### Batch assays experimental setup

In order to test for perturbations of the AD process, 21 anaerobic batch experiments were performed aiming to define the microbial and viral composition in reactors under different conditions. These included addition of mitomycin, temperature shifts, high salt concentration, oxidative stress, pH shifts, and organic overload (details regarding the application of each condition are listed in Additional file 1). Four of these assays involved a combination of temperature change with salt or oxidation stresses. Finally, a control assay was conducted by incubating the inoculum without imposing any stressing condition. All the experiments were performed in triplicate using 300 mL serum glass bottles with a working volume of 50 mL and an organic load of 2 g VS/L, for a total of 66 batches. In addition to the batch experiments, an aliquot of inoculum was saved by storing it at −20 °C immediately after sampling. Prior to incubation, bottles were flushed with nitrogen to achieve anaerobic conditions. Thereafter, the bottles were hermetically closed with butyl rubber stoppers and screw caps. The batch reactors were maintained at 37 °C in a temperature-controlled incubator (BINDER BD260, Tuttlingen, Germany) for 24 h.

### Analytical methods

At the end of each treatment, after 24 h of operation, biogas composition and VFA concentration were measured on all 21 assays plus the control bottles, with the aim of evaluating the effect of the different conditions in the digesters. To determine biogas composition, a gas chromatograph (Shimadzu GC-2014, Kyoto, Japan) equipped with a thermal conductivity detector (TCD) and a packed column (Molecular Sieve 5A, 1.8 m × 2 mm ID) was used. The VFA concentrations were defined with a gas chromatograph (Shimadzu GC-2010 Pro, Kyoto, Japan) provided with a flame ionisation detector (FID) and equipped with fused silica capillary column (30 m × 0.53 mm ID, 1 μm film thickness). The oven temperature was initially set at 50^o^C for 3.5 min, subsequently increased at a rate of 25 °C/min to 130 °C and, finally, increased at a rate of 10^°^C/min until reaching the final temperature of 210 °C, which was maintained stable for 2 min. The temperature in the injection port was 150 °C and in the detector 230 °C. Helium was used as carrier gas for the gas chromatograph.

### DNA extraction and sequencing

**Fig. 1:**
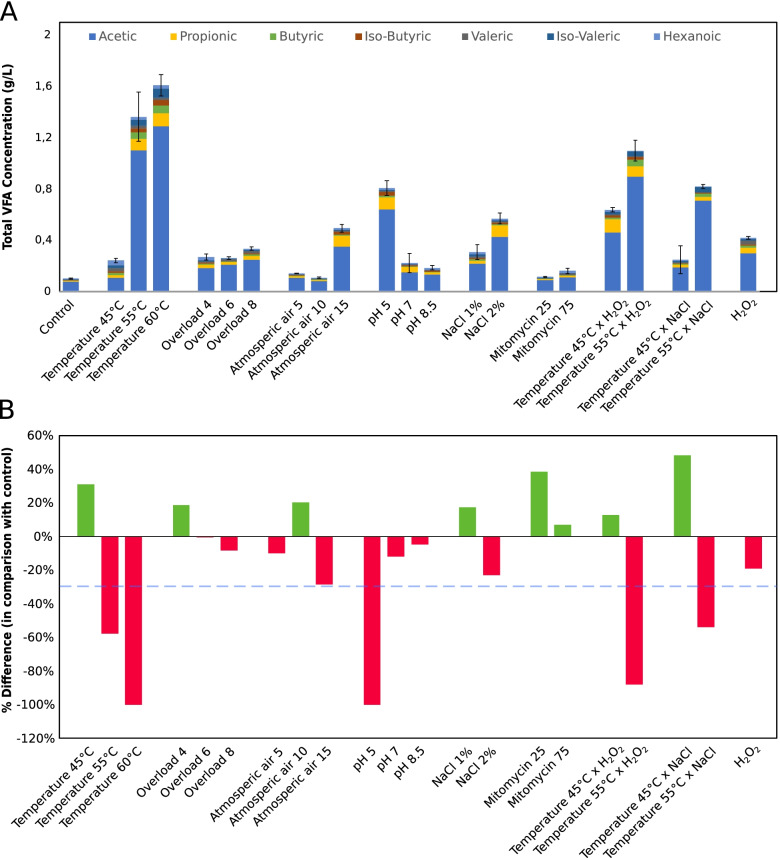
Biochemical measurements of perturbation assays. **A** VFA measurements of bottles after 24 h of perturbation. Error bars represent the standard deviation of the total VFA concentration across triplicate bottles (Additional file 2). **B** Percent difference in methane yield of each perturbation compared to the control assay. The dashed line represents the -30% threshold used to choose which samples to process further

The most promising conditions according to the literature and methane yield variation were selected for DNA extraction, along with the control bottles and the inoculum. Specifically, conditions with a decrease in methane yield between 0 and 30% compared to the control were selected, under the assumption that they were affected enough to potentially observe phage induction, but not to the point of killing the majority of cells (Fig. 1). For each bottle and the inoculum, pellet and supernatant samples were collected as described below for analysing the microbial and viral community, respectively. Centrifugation at high speed was used to separate the viral and bacterial fractions [38, 39]. For the microbial-enriched community (pellet) samples, before starting the extraction, 3 mL of the inoculum were centrifuged at 15000 rpm for 10 min in order to obtain the acquired quantity of 0.2–0.8 g pellet, while the supernatant was discarded. Hereupon, the genomic DNA was extracted. For the viral-enriched community (supernatant) samples, 45 mL of each bottle’s content (or of inoculum from the reactor) were centrifuged (Thermo Scientific SL 16R, New York, USA) at 15,000×g for 10 min at 4 °C. In order to further enrich supernatant samples in viral content, an attempt was performed to filter the supernatant with 0.22 μm syringe filters [40]. However, due to the high content of suspended and dissolved solids of the substrate, filtering was only possible with 1 μm syringe filters (Millex-GP, Merck Millipore Ltd). With the intention of reducing the final volume while using the whole viral content, the filtered flowthroughs were frozen overnight and lyophilised using a freeze dryer (Christ Alpha 1–2, Martin Christ Gefriertrocknungsanlagen GmbH, Germany) coupled with a vacuum pump (rotary vane vacuum pump, Vacuumbrand RZ 2.5, Vacuumbrand GmbH + CO KG, Germany) for 48 h at 0.4 mbar. Before the extraction of the obtained enriched viral community, the lyophilised samples were resuspended in 3 mL PCR water. Subsequently, the samples underwent DNA extraction with DNeasy® PowerSoil® Kit (QIAGEN, Hilden, Germany) following the manufacturer’s protocol. Recovery of DNA from pellet and supernatant samples was ensured by qualitative and quantitative analyses on the samples, using NanoDrop Microvolume UV-Vis spectrophotometer (Thermo Fisher Scientific, USA) and Qubit Fluorometer (Thermo Fisher Scientific, USA). Importantly, DNA yield was limiting for the supernatant samples. Indeed, among all samples, only four tested conditions and the inoculum yielded enough DNA for library preparation, but only upon pooling the replicates for the supernatant. To ensure a coherent comparison, replicates for pellet samples were also pooled before sequencing, and the four conditions and inoculum were further processed.

DNA samples underwent library preparation using the Nextera DNA Flex Library Prep Kit (Illumina Inc., San Diego CA) and were sequenced using the Illumina Novaseq platform at the CRIBI Biotechnology Center sequencing facility (University of Padova, Italy). The sequencing run yielded 6.3 million 150 bp reads on average per sample. Raw data have been deposited at NCBI, BioProject PRJNA767833.

### Assembly and binning

Reads were filtered with Trimmomatic v0.39 [41] and cleaned with BBDuk v38.86. The reads of all pellet and supernatant samples were co-assembled using MEGAHIT v1.2.9 [42]. The quality of the co-assembly was assessed with QUAST v5.0.2 [43]. The filtered and cleaned reads were then mapped back on the assembly with Bowtie 2 v2.3.5.1 [44]. Details and parameters used for these programs are reported in Additional file 1. The assembled contigs were analysed with PPR-Meta v1.1, CheckV v0.7.0, VIBRANT v1.2.0 and the PHASTER web server [23, 24, 27, 28]. CheckV results were filtered excluding predictions with “not determined” quality and no viral genes detected. PPR-Meta predictions were filtered for viral scores of 0.75 or higher. VIBRANT and PHASTER predictions were carried forward with no pre-filtering. A first list of contigs classified as viral was defined by considering predictions made by either PHASTER alone, or at least two of the other programs (Additional file 1: Figure 1).

A binning procedure was performed with MetaBAT2 v2.12.1 [45, 46] using a minimum bin size of 10,000 bp. Here, we refer to the output of the binning algorithm as “bins” and to bins which have passed quality control and are thus considered representative of prokaryotic genomes as “MAGs”. The bins yielded by MetaBAT2 were evaluated and divided into Metagenome-Assembled Genomes (MAGs), viral MAGs and unclassified contigs, according to a procedure described in Additional file 1. For prokaryotes, bin quality, completeness and contamination were measured with CheckM v1.1.2 [47]. Finally, CheckV was run again on the viral MAGs recovered from binning in order to calculate genome quality and completeness.

### Coverage profiles

Relative abundance of prokaryotic and viral MAGs was calculated by performing genome count per million (CPM) normalisation, which takes into account genome length and sequencing depth, on read counts obtained from the reads mapped on the assembly. The values obtained were highly similar with those obtained using CheckM software v1.1.2 (Additional file 1: Figure 2). For the 50 most abundant MAGs, the coefficient of variation, defined as the standard deviation divided by the mean, was calculated. The mean was calculated across pellet samples.

The effect of the different shocks was evaluated by calculating the log ratio between the relative abundance of genomes in each shock and the mean relative abundance across all the conditions considered. In this context, a positive log ratio refers to an abundance higher than average and vice versa. For an overall comparison of the conditions, Spearman correlation was calculated between log ratio values in different samples. The calculation of the Spearman correlation coefficients and the corresponding *P*-values was carried out with SciPy v1.3.1 [48]. The same analysis was performed by using the control sample as a reference.

The 50 most abundant MAGs and viral genomes were clustered by computing the Euclidean distance from the log ratios under different conditions and using an average linkage method. Correlation values between MAGs were computed by using all eight treated samples (four pellet, four supernatant). The software SparCC (commit 2ddc13f, February 2020) was used to calculate correlation coefficients while taking into account the compositional nature of the data [49]. The input was a matrix of mapped reads on each genome in the eight samples. Significance of the obtained correlation values was assessed by generating 1000 bootstraps and calculating two-sided pseudo *P*-values.

### Taxonomic assignment and functional annotation

Prokaryotic MAGs were taxonomically assigned using GTDB-Tk v1.4.1 and converted to NCBI taxonomy with the script gtdb_to_ncbi_majority_vote.py [50]. Viral genomes were assigned via Hidden Markov Model against the Prokaryotic Virus Orthologous Groups (pVOGs) database using hmmsearch from the HMMER v3.3.2 suite [51, 52].

ORFs were predicted using Prodigal v2.6.3 [53]. Taxonomy was assigned on the basis of a consensus rule, as previously reported [21]. Taxonomy assignment is explained in detail in Additional file﻿ 1. Prodigal was also used to detect the presence of alternative stop codons in viral sequences, following the method used by Borges and colleagues [54].

Functional annotation was carried out on protein encoding genes predicted on prokaryotic and viral genomes using the eggnog-mapper server [55]. The completeness of KEGG modules in each microbial genome was calculated with KEMET [56]. Furthermore, ORFs annotated with KEGG orthologs belonging to putative alternatives to the Wood-Ljungdahl pathway (WLP) were counted in each MAG to identify potential syntrophic acetate oxidising bacteria. Proteins involved in carbohydrate hydrolysis were searched against the dbCAN database [57] using hmmsearch and annotated. Proteins were also analysed with gutSMASH [58], in order to find gene clusters related to VFA production and metabolism. Fisher’s exact test, implemented in SciPy v1.3.1, was employed in order to assess whether the occurrence of the GH33 enzymatic family was significantly higher in viral genomes than in microbial genomes.

### Detection of induction in integrated prophages

With the aim of evaluating the induction of putative integrated phages, an analysis was carried out on the eight samples analysed in this work and extended to 110 samples of the AD database [2]. The aim of the analysis was to check whether prophages detected in the induction experiment reported in this study were also present (and, possibly, induced) in other, unrelated AD communities. MAGs featuring integrated viruses were split into viral and nonviral sequences by extracting the viral sequence predicted within MAGs. This approach resulted in a dataset of 64 prokaryotic MAGs and 64 corresponding integrated viral sequences. Ten million reads were randomly extracted from fastq files and mapped on the database generated from the extracted prophages and MAGs using Bowtie 2. Genome coverage of MAGs and prophages in the different samples was calculated with CheckM coverage. One coverage value was obtained for each contig. Coverage values for each genome were obtained by averaging the values of individual contigs. The coverage threshold for a species to be considered was set to 0.01. The virus/MAG coverage ratios were calculated and the distributions of their values across samples and across genomes were inspected. Finally, log ratios were clustered with an average linkage algorithm based on Euclidean distance. Prophages were considered putatively induced in a sample when the log ratio was greater than 10. This threshold was chosen to exclude values resulting from noise, based on the exponential-like distribution which reaches a plateau around the value of 10 (Additional file 1).

## Results

### Anaerobic digestion perturbation assays

The current study investigated the effect of environmental stresses on viral and microbial composition of AD communities present in laboratory-scale reactors. The experimental plan included 21 different environmental perturbations known to affect the AD microbial community and to stimulate the induction of integrated prophages (Additional file 1) [14, 59–65]. Some of these conditions occur in biogas plants and negatively impact the reactor performance, but they do not completely disrupt the microbial community, and methane production can be recovered if the conditions are removed. Overall, the highest decreases in methane yield were observed with low pH and temperature shifts to 55 °C or 60 °C (Fig. 1). Temperature shifts had a similarly strong effect if combined with high salt concentration and a much stronger one when paired with oxidation. A moderate temperature increase, on the other hand, registered positive effects on the production of methane, even when combined with other factors. Interestingly, the treatment with mitomycin did not result in a reduction of methanogenesis. In fact, for both concentrations of mitomycin assessed, the methane yield increased. Importantly, the aim was to identify the perturbations where viral induction was more likely to be detectable. A condition for this was that the microbial community had to be perturbed, but not completely disrupted. The most promising batches, either according to data found in literature, or according to the variation in methane yield, were carried over to DNA extraction and sequencing. DNA yields for the viral fraction were often exceptionally low, despite using all the available volume to perform the extraction. In several cases, including the control bottle, the insufficient yield of viral DNA made it impossible to generate sequencing libraries or provided raw reads of low quality that were discarded (data not shown). Ultimately, four conditions were successfully sequenced and analysed. All of them are characterised by a moderate decrease in the methane yield (up to −30%) with respect to the control bottle. These were organic overload of 8 g VS/L, exposure to atmospheric air at a concentration of 15 mL O_2_/g VS, pH increase to the value of 8.5, and exposure to H_2_O_2_ at a concentration of 3 mM. The fourth condition, which obviously does not occur in biogas plants, was set up in order to mimic a strong oxidative shock, possibly happening during a massive oxygen influx in the system.

### Viral and microbial community

Metagenomic analysis allowed the recovery of 1,092 viral genomes (virMAGs). A parallel binning approach recovered 120 microbial MAGs, 72 of them being of high quality according to MIMAG standards. It is reported in the literature that about half of known bacterial genomes feature integrated prophages in their sequence [66]; similarly, 64 of the 120 MAGs identified in this study harbour prophages (Additional file 3).

**Fig. 2:**
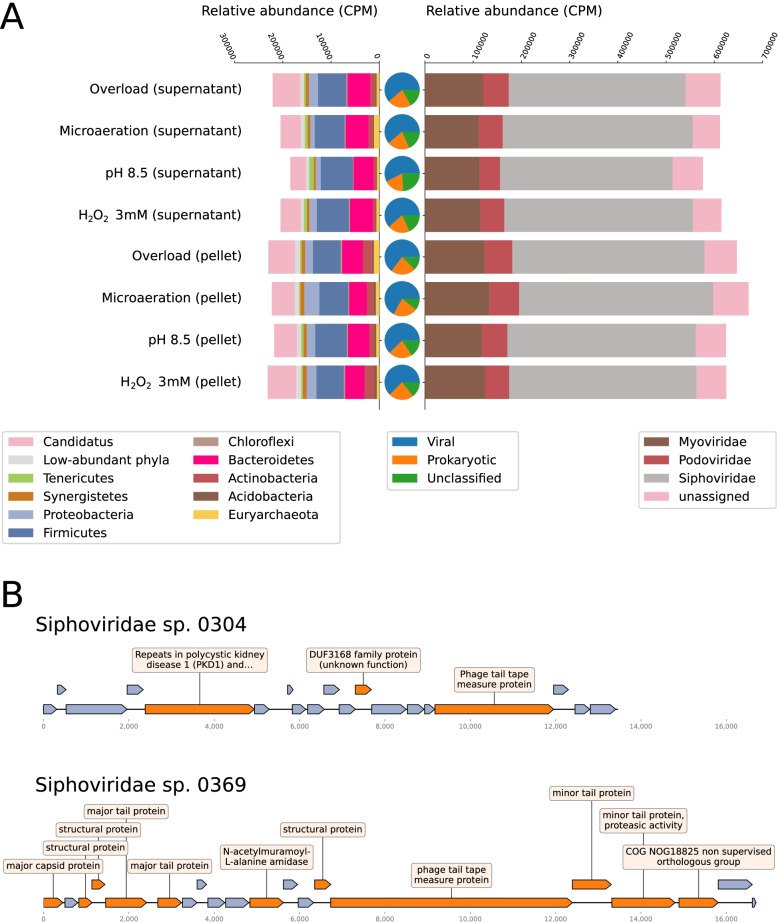
Overview of the prokaryotic and viral community. **A** Relative abundance of prokaryotic phyla (left) and viral families (right), expressed in genome count per million of genomes (CPM) in each sample. The pie charts in the middle represent the overall CPM abundance of viruses (blue), prokaryotes (orange) and unclassified (green) contigs. The term “Candidatus” in the legend refers to the sum of all the candidate phyla. **B** Genome map of two significant viruses retrieved in this study. Annotated ORFs are depicted in orange; uncharacterised ORFs in blue

The normalised relative abundance of the viral component is very high, reaching almost 70% of the total community (viral + microbial) in some samples (Fig. 2). Viral MAGs and single-scaffolds viral genomes ranged in size from 1502 to 195,329 bp, covering the vast majority of the sequence lengths space occupied by prokaryotic viruses with the exclusion of jumbophages [67] (Additional file 3). This was done by design, as the three viral bins longer than 200 kpb were divided into single contigs (Additional file 1). Furthermore, CheckV showed that these bins featured a low completeness and high contamination, justifying the approach taken in their regard. The combined approach of viral prediction and binning yielded a total of 16 high-quality and five complete viral MAGs according to MIUViG standards [68]. Other metaviromic studies report similar numbers of high quality viral genomes per dataset [9, 21, 69].

The DNA virome is largely dominated by *Caudovirales* phages belonging to the *Myoviridae*, *Podoviridae* and *Siphoviridae* families, confirming similar results observed in previous studies investigating AD viromes [9, 11]. A few small contigs (< 5 kb) were classified as *Inoviridae* or *Microviridae*. This is consistent with the fact that species within these taxa tend to have very small genomes, in the order of kilobases. As they are short and underrepresented in databases, it is difficult both to detect them in metagenomes and to estimate completeness and contamination. Among the viral sequences investigated (considering all viral MAGs and prophages integrated in the prokaryotic MAGs), 4.5% were unequivocally assigned at species level. Furthermore, the vast majority (88%) of the viral genomes were assigned at family level.

The distribution of relative abundances among viral genomes is very skewed, with a few prominent viruses (including *Siphoviridae* sp. 0304, *Siphoviridae* sp. 0142, *Virus* sp. 0026, *Siphoviridae* sp. 0307; Additional file 1: Figure 3) and the majority having very low values. In particular, *Siphoviridae* sp. 0304 (Fig. 2B) is by far the most abundant, with a relative abundance of about 5.3% on average across samples.

Single-scaffold phage genome *Siphoviridae* sp. 0431, while not being as abundant as the virMAGs previously mentioned, is a prominent component of the viral community, with an average abundance of 5400 CPM (0.54%) across samples, which spikes at 13,009 CPM in the supernatant part of the sample subjected to alkaline condition.

The prokaryotic composition is consistent with the results of previous works describing anaerobic digestion communities [70, 71]. *Firmicutes* was the most abundant bacterial phylum (25–30% in relative abundance) followed by *Candidatus* Cloacimonetes (20%), *Bacteroidetes* (18%) and *Proteobacteria* (9.4%) (Additional file 3). Four of the 120 prokaryotic MAGs were classified as *Archaea*, accounting for 3 to 4% of the microbiome (Additional file 3). This set is represented by: *Methanoculleus* sp. 0064, *Methanothrix* sp. 0024, *Methanosarcina flavescens* 0114 and *Methanosarcina mazei* 0049. *Methanoculleus* species perform hydrogenotrophic methanogenesis [72, 73], archaea of *Methanothrix* genus perform aceticlastic methanogenesis, while the *Methanosarcina* are generalists [74]. The two *Methanosarcina* identified in this work harbour integrated viral sequences, belonging to the *Siphoviridae* family. The two genomes *Candidatus* Cloacimonetes spp. 057 and 073 are highly abundant, accounting for a substantial percentage of the bacterial community, from 16% in the pH 8.5 supernatant sample up to 23% in the organic overload supernatant sample. Other examples of AD communities dominated by members of *Candidatus* Cloacimonetes, not included in the AD database, have been described in literature [75–77]. Members of this phylum have been suggested as glycolytic in previous studies [78].

### Effect of tested conditions on the metagenome

**Fig. 3:**
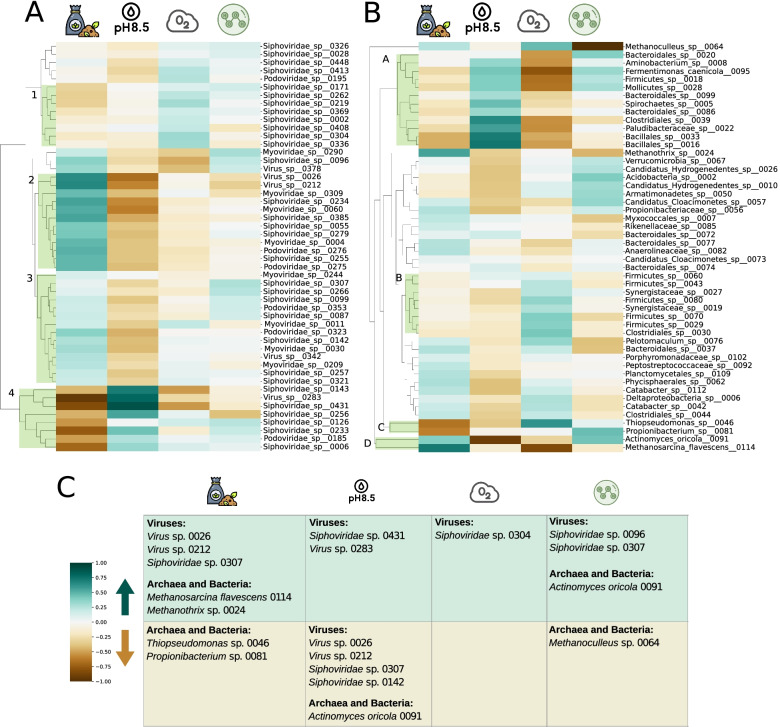
Heatmaps displaying the log ratios of MAGs and virMAGs relative abundance in each condition over their mean relative abundance across samples. **A** The 50 most abundant viral MAGs are displayed, and the average abundance is calculated on the supernatant samples. Four clusters are highlighted: cluster “1” is exclusively composed of *Siphoviridae* phages undergoing a mild increase during microaeration; cluster “2” is characterised by a marked increase during overload as well as a decrease in pH; cluster “3” is similar to cluster “2”, but the variations are less evident; cluster “4” is composed of phages sharply decreasing during overload and increasing when exposed to basic pH. **B** The 50 most abundant prokaryotic MAGs are displayed, and the average relative abundance is calculated across pellet samples. Coherent behaviours in terms of response to conditions are depicted as clusters: cluster “A” comprises MAGs increasing in relative abundance when exposed to basic pH and negatively affected by microaeration; cluster “B” is composed of MAGs with slightly increased average relative abundance under microaeration; in cluster “C” and cluster “D”, respectively, a sharp decrease and increase during overload treatment are evidenced. **C** The summary table contains some functionally relevant species discussed in the main text

The effect of the different treatments on MAGs and viruses was evaluated by calculating the log ratio of the relative abundances with respect to the average value (see the “Materials and methods” section). The same analysis was performed by comparing the treated samples with the inoculum, the results of which are reported in Additional file 1: Figure 4 due to the marked differences in the microbial profiles existing between the control and the other conditions. A hierarchical clustering performed on the most abundant microorganisms and viruses highlighted groups of species with similar behaviours (Fig. 3). Correlation values between all genomes were calculated considering the compositional nature of the data and are reported in Additional file 3.

Groups of phages with very definite behaviours emerged from the hierarchical clustering (Fig. 3). These groups are heterogeneous in terms of viral taxonomies, yet certain genes are characteristic of each group. The 50 most abundant viral genomes match against a total of 371 HMM profiles of the pVOG database. Of these, 266 (72%) are cluster-specific. It is evident that basic pH and overload tend to have opposite effects on phages (Spearman’s *⍴* = − 0.28, *p* < 0.05), particularly in clusters “2” and “4”. The former is characterised by eight phages which decrease in relative abundance during overload treatment (log ratios between − 0.5 and − 1, see Additional file 3). Three of these genomes have an increase in relative abundance only when exposed to basic pH, with *Siphoviridae* sp. 0431 and *Virus* sp. 0283 almost doubling. It should be noted that increased abundance could have different biological explanations including phage induction and increased abundance of the host. In the latter cluster, the opposite effect can be seen, and in particular two genomes (*Virus* sp. 0026 and *Virus* sp. 0212) are those most heavily affected by basic pH (log ratios − 0.68 and − 0.59, respectively) and overload (log ratio 0.64 and 0.66, respectively). Viruses of cluster “3”, similarly, increase in abundance during organic overload and decrease when exposed to basic pH, but less markedly (average log ratios 0.20 and -0.31, respectively). This cluster includes *Siphoviridae* sp. 0307 and *Siphoviridae* sp. 0142, which are the second and third most abundant viruses present in the dataset. The Yersinia outer protein gene (*yopX*), which is likely to be involved in life cycle regulation in temperate bacteriophages [79], is only present in genomes belonging to cluster “3”, chiefly *Siphoviridae* sp. 0142, contributing to the hypothesis that these viruses are temperate. The main dominant viral genome, *Siphoviridae* sp. 0304, is part of cluster “1”, entirely composed of *Siphoviridae* and the only cluster of genomes whose relative abundance increases during microaeration (log ratios from 0.11 to 0.31). Cluster “1” is the only one where matches against pVOGs VOG3653, VOG3654, and VOG9328 were found, all of which are annotated as tail proteins. The ORFs matching against these HMM profiles are always found in a duo: one matches with VOG3653, the other both with VOG3654 and VOG9328 (Fig. 2B). This result suggests that the proteins encoded by these two genes are complementary tail components. Overall, basic pH and overload affect phages the most. Basic pH is the condition with the most negative influence on the viruses considered, while overload is mostly associated with positive log ratio values. Unexpectedly, exposure to H_2_O_2_ is not responsible for great variations in relative abundance of phages. Considering the 50 most abundant phages, most log ratio values under hydrogen peroxide exposition fall between − 0.1 and 0.1, and only a small set of viruses shows a mild increase, including the aforementioned *Siphoviridae* sp. 0096 and *Siphoviridae* sp. 0307.

Microaeration and exposure to hydrogen peroxide have strong effects on MAGs and seem to have opposite effects on the whole microbial community, displaying a slight anticorrelation (Spearman’s *⍴* = − 0.40, *p* < 0.005). Basic pH is another condition that predominantly affects the microbial community. Genomes in cluster “A” (Fig. 3), sharply increase in relative abundance under basic pH and decrease under microaeration. This cluster shows a relative lack of integrated proviruses in the MAGs’ genomes: only two MAGs out of 14 include viral sequences (14%), whereas in the entire dataset, 64 MAGs out of 120 do (53%). Contrariwise, in cluster “B” 6 out of 8 MAGs carry integrated prophages. However, the small number of genomes made it difficult to draw statistically sound conclusions regarding these differences.

The behaviour of the three archaeal MAGs included in the analysis is peculiar and, although they end up in distant places in the clustering (Fig. 3), they have common characteristics. First, they do not cluster with bacterial genomes, but rather exhibit a unique behaviour. Secondly, their relative abundance varies markedly (coefficients of variation between 0.34 and 0.46, higher than 40 MAGs out of the 50 included in the heatmap), evidencing a marked response to the changes in the experimental conditions. For example, *M. flavescens* 114 and *Methanothrix* sp. 24 show a marked increase under organic overload, with log ratios of 0.71 and 0.56, respectively.

Another common trend of archaeal MAG abundance in relation to shocks is a decrease in response to H_2_O_2_ exposure. This is coherent with biochemical measurements, as the sample is characterised by the highest concentration of acetate and VFA, and a substantially lower methane yield (Additional file 2, Fig. 1). A kinetic imbalance between acid producers and consumers, reflected by low methane production, is revealed by VFA accumulation [62, 80]. This sample shows the highest concentration of VFA, including acetate. This is coherent with the idea that a halt in the activity of acetoclastic archaea leads to an accumulation of acetate and a decrease in methane production.

Two isolated small clusters dubbed “C” and “D”, comprising two MAGs each, comprise species which display marked variations in relative abundance among different conditions. Cluster “C” comprises *Thiopseudomonas* sp. 046 and *Propionibacterium* sp. 081, both decreasing with organic overload, but the former sharply increased with air. The *Thiopseudomonas* genus has been described as a facultative anaerobe, catalase- and oxidase-positive [81]. Furthermore, five ORFs in the two *Thiopseudomonas* MAGs are functionally related to oxidative stress (EC numbers 1.11.1.6, 1.11.1.15, and 1.8.1.9, Additional file 4), which could explain a possible air tolerance. Cluster “D” comprises *Actinomyces oricola* 091 and *M. flavescens* 114. The bacterium is negatively affected by basic pH, with its relative abundance almost halved with respect to the mean (mean abundance 6,631 CPM; abundance in basic pH 3541 CPM; log ratio − 0.90). Its relative abundance also suggests an increase during H_2_O_2_ exposure.

Finally, *Pseudomonas formosensis* 084 and *Thiopseudomonas* sp. 083 display extreme variation between conditions. Their relative abundance is exceptionally low under overload (log ratios -4.8 and -2.2 respectively) which is their most striking characteristic. Furthermore, both revealed a higher relative abundance under microaeration, and a lower relative abundance with H_2_O_2_ in comparison to the average value across conditions.

### Functional categories of proteins encoded in MAGs and viral MAGs

The tested conditions appear to have an important effect in shaping the structure of both the viral and microbial communities. Functional annotation was employed to investigate the link between the variation in community composition and the tested conditions. The AD process is carried out by a multitude of microorganisms, each one playing a number of roles in the degradation and conversion of organic matter [82]. A starting point for the metabolic characterisation is the functional annotation of genes. Analysis of the functional categories was performed on protein-coding genes identified in all predicted viruses and microbial genomes. Notably, 70% of the ORFs encoded by prokaryotic genomes registered a match in the KEGG Orthology database, while this percentage is as low as 30% in viral genomes. Viruses, although they do not perform metabolic activities in the community, can influence and modulate microbial functionality via infection, induction, and HGT. It was recently reported that, in the design of synthetic microbial communities, it is of utmost importance to determine the absence of integrated, putative inducible prophages, to ensure the stability of the process [83, 84]. According to this approach, the presence of prophages was verified, in order to determine the putative level of vulnerability of specific steps of the AD process. AD is divided into four main steps: hydrolysis, acidogenesis, acetogenesis, and methanogenesis [85]. In the hydrolysis step, complex organic molecules are broken down to their monomers, which are then converted into VFAs by the guild of acidogenic bacteria. VFAs are then employed by the acetogenic species in the production of acetate, hydrogen and CO_2_, upon which the methanogenic archaea feed, producing methane. In an attempt to categorise the MAGs according to their role in the AD process, particular attention was paid to gene categories that are important in each of these steps. These gene categories were grouped as follows: (I) genes involved in binding and degradation of polysaccharides, especially cellulosome-related protein families; (II) genes related to VFA production or metabolism; (III) genes pertaining to the Wood-Ljungdahl pathway (WLP) or pathways proposed as alternatives and potentially involved in syntrophic acetate oxidation; and (IV) genes involved in methanogenesis (Additional file 4).

Viruses have a double role in the funnel-shaped web of interaction of the AD. On one hand, they may carry genes conferring additional enzymatic functions to their hosts; on the other hand, they represent a threat to the host, as their lifestyle often involves the hijacking of the host metabolism and its death. For this reason, genes of interest were searched in the prophages integrated in the aforementioned MAGs, both with a positive and a negative impact on the host metabolism. Free viral genomes were also investigated as they could represent temperate viruses.

**Fig. 4:**
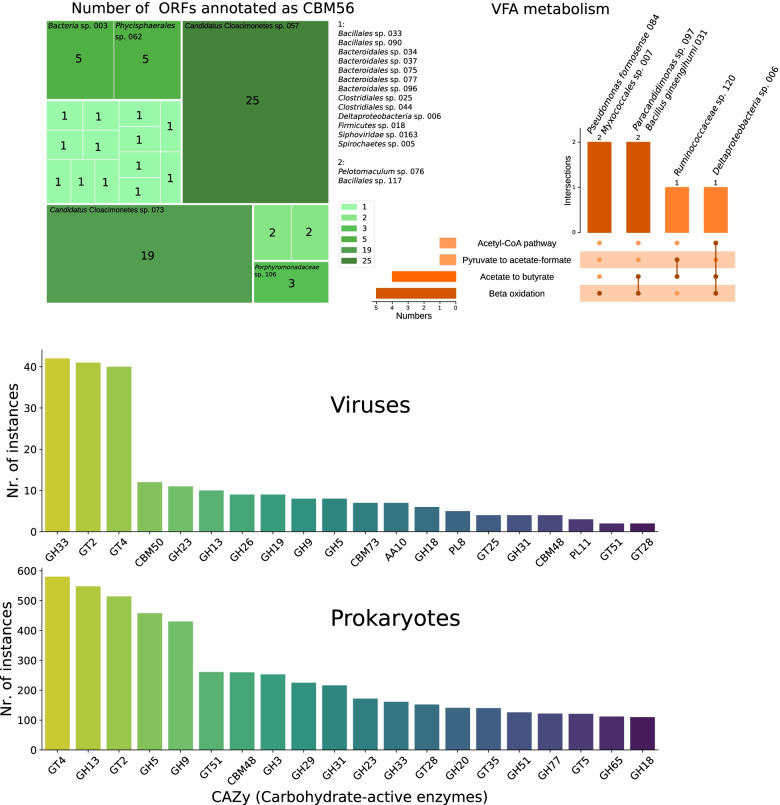
Distribution of metabolic genes in the microbiome. **A** Tree map showing the number of ORFs annotated as CBM56, a family of cellulose-binding enzymes, in each genome. More than half of the CBM56 ORFs of the whole dataset are found in the two *Candidatus* Cloacimonetes MAGs. **B** Upset plot summarising the MAGs coding for VFA-related metabolic pathways. The horizontal bar plot indicates how many species carry out each process, the vertical bar plot shows how many and which pathway each species codes for. **C** The 20 most frequent families of carbohydrate-active enzymes of the CAZy database both in viral (top) and prokaryotic genomes (bottom)

The MAGs encoding the largest number of ORFs annotated with carbohydrate-binding functions (guild I) belong to the candidate phyla Cloacimonetes and Hydrogenedentes. The two *Candidatus* Cloacimonetes genomes are characterised by the occurrence of ORFs annotated as CBM56 by dbCAN (Fig. 4A). This enzymatic family is associated with a beta-1-3-glucan binding function; hence, it is probably involved in favouring the binding of *Bacteria* to cellulose substrates. A glycolytic role has been suggested for *Candidatus* Cloacimonetes bacteria in a previous study [78]. Moreover, *Candidatus* Hydrogenedentes sp. 010 and *Candidatus* Hydrogenedentes sp. 026 feature, respectively, 13 and 27 genes annotated as dockerins, i.e., proteins that take part in the formation of cellulosomes.

Genes encoding enzymes related to carbohydrate synthesis, degradation and binding were found in 18 out of 64 integrated viral genomes. Three enzymatic families largely outnumber the others: GH33, GT4 and GT2 are found in 42, 41 and 40 genomes respectively, with each single family representing more than 15% of the 262 hydrolytic enzymes found in viral genomes.

GT4 and GT2 are glycosyl transferase families and are respectively the first and the third most frequent enzymatic families found in prokaryotic genomes, with 580 and 514 matches out of a total of 7073. It is known that mobile genetic elements can provide novel metabolic functionalities to their hosts [86].

GH33, instead, is a family of sialidases and neuraminidases, and is represented by 161 ORFs among prokaryotic sequences, which ranks it as the twelfth most represented. It is thus more frequent in viruses than in prokaryotic sequences (*p* = 10^−22^, Fisher’s exact test), hinting at their importance in the viral physiology (Fig. 4C).

The presence of genes related to VFA metabolism and acetate formation (guild II) was evaluated by checking the completeness of the beta-oxidation module and the presence of a selection of clusters of genes [58]. The beta-oxidation module (M00087) was complete in five genomes (Additional file 4). Three of these genomes, as well as *Ruminococcaceae* sp. 120, encode enzymes involved in acetate, pyruvate and butyrate metabolism (Table 1). In particular, the genome of *Deltaproteobacteria* sp. 006 features the complete M00087 beta-oxidation module, genes belonging to the “Acetate to butyrate” and “Acetyl-CoA pathway” metabolisms and an integrated prophage of the *Myoviridae* family (Table 1).

**Table 1:** *Bacteria* related to VFA metabolism. The table reports the phylum of belonging, the family of integrated prophages, the completeness of the beta-oxidation KEGG module (M00087) and the presence of relevant metabolic gene clusters detected using the gutSMASH software

Genome ID	Phylum	Prophage	M00087	Metabolic gene clusters
*Deltaproteobacteria* sp. 006	*Proteobacteria*	*Myoviridae*	Complete	3 acetate to butyrate; 1 acetyl-CoA pathway
*Myxococcales* sp. 007	*Proteobacteria*	Absent	Complete	Absent
*Pseudomonas formosensis* 084	*Proteobacteria*	*Myoviridae*	Complete	Absent
*Paracandidimonas* sp. 097	*Proteobacteria*	*Siphoviridae*	Complete	3 Acetate to butyrate
*Bacillus ginsengihumi* 031	*Firmicutes*	*Siphoviridae*	Complete	3 Acetate to butyrate
*Ruminococcaceae* sp. 120	*Firmicutes*	Absent	Incomplete	1 Pyruvate to acetate-formate; 3 acetate to butyrate

*Bacteria* potentially involved in syntrophic acetate oxidation (guild III) were identified by evaluating the presence of genes belonging to the Wood-Ljungdahl pathway (WLP) or its putative alternatives, the glycine synthase-reductase pathway (GSRP), and the reductive glycine pathway (RGP). In this dataset, these alternative WLP modules seem to be exclusive of *Firmicutes* (Additional file 4). Eleven MAGs comprise genes belonging to oxidative pathways, ten of which belong to *Firmicutes* (e.g. *Firmicutes* sp. 0060 and *Catabacter* sp. 0112) and one to *Chloroflexi* (*Anaerolineaceae* sp. 0082). *Firmicutes* bacteria have already been reported as capable of converting acetate to CO_2_ through the reverse WL pathway [87, 88]. Furthermore, gene annotations in previous works [89] have already identified bacteria from the *Chloroflexi* phylum as potential syntrophic acetate oxidising bacteria. In *Firmicutes* sp. 0060, both the glycine cleavage system and the GSRP were fully complete, while the RGP was 83% complete. A noteworthy viral genome recovered is *Siphoviridae* sp. 0243, which is an 84-kb phage with an estimated completeness between 80 and 100% and harbours a section of the glycine synthase-reductase pathway (GSRP). This suggests that this virus can confer additional enzymatic capabilities to its host, giving it an alternative to the Wood-Ljungdahl pathway.

Methanogenesis (guild IV) is exclusively carried out by methanogenic archaea. This guild is represented by a heterogeneous population of hydrogenotrophic (*Methanoculleus* sp. 0064), acetoclastic (*Methanothrix* sp. 0024) and generalist methanogens (*M. flavescens* 0114; *M. mazei* 49). The two MAGs assigned to *Methanosarcina* genus have integrated *Siphoviridae* proviruses.

### Evaluation of selected prophages abundance in the AD database

Given the wide presence and importance of integrated proviruses across all metabolic guilds, the search was broadened by considering 123 additional shotgun sequencing experiments deposited in public AD databases [2]. Many of these experiments investigated AD reactors operating under a variety of parameters including temperatures ranging from 35 to 55 °C, different feedstocks and stressful conditions such as lipids overload [90] or high concentration of ammonia [91]. For each MAG featuring integrated proviruses, the viral/host ratio was defined as the ratio between the read coverage of the viral and the non-viral components. This proportion was calculated separately for each experiment, with the aim of showing whether a specific prophage increases in abundance with respect to the host under specific environmental conditions.

### Microbial and viral diversity across the AD database

In the samples from the present study, the average prophage/host ratio was equal to 1.1 and the maximum value was 13.3. Contrariwise, considering all experiments from the AD database, the average ratio rises to 12.0 and the maximum is over 4200 (Additional file 5). These data reflect the diversity of environmental conditions across the AD database and underline their importance in shaping the microbial and viral community. The read coverage across the whole dataset shows that viruses and hosts are not always present in the same community. There are 64 MAGs featuring integrated proviruses, on which reads from the additional experiments were mapped, resulting in 7872 values of coverage ratio. In 2722 cases (34.6%) the integrated virus was not found, despite the presence of the host (Additional file 5). The opposite is much rarer: only in 22 cases (0.3%) reads map only onto the viral part of the MAG. These cases may be explained by the ability of the viral species itself or related strains to infect a different host [92]. In most cases (1221 occurrences, 15.5%) where the host microbe is not present, the bacteriophage is not present either.

### Effects of temperature

**Fig. 5:**
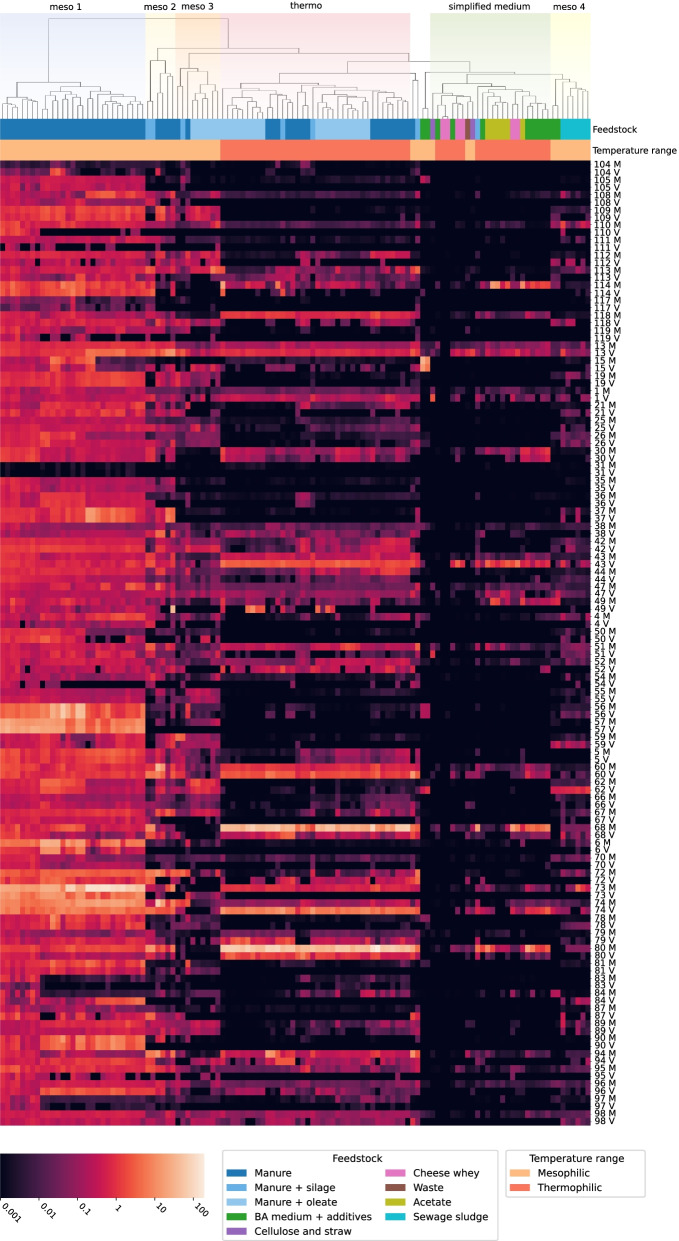
Coverage of the MAGs with integrated phages in the 8 samples from the present experiment and in 110 samples from the AD database. The colour scale of coverage is logarithmic. The sample names are reported in Additional file 5 in the order in which they appear in the heatmap. MAGs are identified by the same sequential numbers that appear in their complete name (e.g., “*Firmicutes* sp. 043” is here “43 M”). “M” denotes the prokaryotic fraction of the MAGs, while “V” indicates the integrated viral part. Feedstock and temperature range are displayed as coloured labels at the top of the heatmap. Samples are grouped into clusters based on Euclidean distance. Clusters that are relevant to our analysis are highlighted at the top of the image with pastel colours

Temperature is the strongest driver of clustering: most of the mesophilic samples end up in four sub-clusters of respectively 29, 6, 9 and 8 sequencing experiments (Fig. 5). One large cluster is entirely composed of thermophilic samples. Here, 73-98% of MAGs are still present, although their coverage is often lower than those registered in our samples. However, only 26–53% of the respective phages are present and the prophage/host ratio is 7.74 on average.

In order to evaluate the impact of the temperature in the composition of the viral community, the coverage of each provirus in mesophilic samples was compared with the coverage in thermophilic samples with a Mann-Whitney *U* test. Out of 64 proviruses, 54 (84%) are more abundant in the mesophilic samples, and this finding is expected because this study was performed on mesophilic reactors. However, four outliers were found to be more prevalent in thermophilic groups (*p*-value threshold = 0.05, Table 2), and six do not show significant differences.

**Table 2:** Proviruses which score a higher read coverage in thermophilic samples, as confirmed by Mann-Whitney *U* tests

Provirus name	Taxonomy	***p***-value
provirus *Firmicutes* sp. 043	*Siphoviridae* sp.	1.5e−11
provirus *Firmicutes* sp. 060	*Siphoviridae* sp.	4.5e−2
provirus *Bacteroidales* sp. 074	*Myoviridae* sp.	2.9e−2
provirus *Firmicutes* sp. 080	*Siphoviridae* sp.	3.5e−05

### Impact of simplified feedstocks

A 26-sample cluster named “simplified medium” is characterised by the predominance of samples in which the feedstocks have a controlled or restricted composition: out of 26 reactors, 10 use as feedstock BA medium mixed with simple components as carbon sources, such as acetate, glucose, avicel, and VFA mixtures; 6 samples were fed with cheese whey 6 with acetate as the major substrate (Fig. 5). The peculiarity of this cluster is that they have a low number of MAGs and phages in common with our samples. On average, half of the MAGs identified in this study are present in this cluster, with two samples having as few as 6 and 9 MAGs. This is also due to the smaller number of taxa identified in the community present in these samples, which amplifies the difference with the samples investigated in this study. The integrated prophages are detected on average for 10% of MAGs, with only one sample having more than 20%.

The most ubiquitous MAG-prophage couples are *Clostridiales Family XIII Incertae Sedis* sp. 013, *Firmicutes* sp. 043 and *Bacteroidales* sp. 047, with their respective prophages. These MAG-prophage couples are found in association in over 90% of the samples.

The three least frequent MAG-prophage couples are *Bacillales* sp. 117, *Bacillales* sp. 090 and *Candidatus* Cloacimonetes sp. 057, all of them predominantly present at mesophilic conditions. Despite the prevalence of *Candidatus* Cloacimonetes sp. 057 in the data gathered in this study as mentioned earlier, this MAG is exclusively found in studies belonging to the group “meso 1”, characterised by mesophilic temperatures and cattle manure as feedstock (Fig. 5).

### Behaviour of proviruses

Putatively induced phages in the different AD metagenomes have been estimated by using as a proxy the ratio between the coverage of the provirus compared with the rest of the MAG. A provirus has been considered induced in a certain condition when the ratio was larger than 10, based on the cumulative distribution of the viral/host abundance ratios (Additional file 1: Figure 5). However, the rise in abundance of a virus with respect to its host could be explained by factors different from induction, such as the presence of an alternative host or a low number of reads mapped on both genomes, and this bias should be taken into account when discussing this aspect.

In the “simplified medium” cluster, 13 out of the 24 MAG-virus couples have an average ratio indicating an induction. Most of the MAGs belong to the *Bacteroidales* and *Clostridiales* orders, with the exceptions of three members of *Firmicutes*, *Acholeplasmatales* sp. 079 and *Candidatus* Hydrogenedentes sp. 066. The latter is hypothesised to play a role in the hydrolysis of cellulose, and, as such, it is found as induced in a sample fed with cellulose and straw (virus/host coverage ratio = 94).

Provirus *Firmicutes* sp. 043, which infects one of the most ubiquitous MAGs, has an average coverage ratio of 493 in the “simplified medium” cluster and of 68 in the “meso 4” cluster. Provirus *Peptococcaceae* sp. 118 has an average ratio of 165 and 368 in the “meso 3” and “meso 4” clusters, respectively. Provirus *Pseudomonas formosensis* 084, whose host participates in the degradation of fatty acids via beta-oxidation, is detected as induced in the “meso 4” cluster, where it is present in five out of eight samples, and its coverage ratios range from 27 to 2,779. This cluster is characterised by mesophilic temperatures and most of the reactors in it are fed with sewage sludge.

Provirus *M. mazei* 049 scores a read coverage more than 10 times higher than its host in 9 samples belonging to the “thermo” cluster. In one sample, the virus has a coverage of 37, whereas the host only has 0.05. This is most likely a case in which the virus itself is present but infecting a different host.

Overall, the variety of environmental conditions under which AD occurs provides a range of opportunities to explore the interactions between viruses and their hosts, revealing large-scale trends which would otherwise be difficult to detect.

## Discussion

In this study, the virome of AD communities undergoing several prophage-inducing stresses was investigated. As expected, much of the viral diversity hereby explored is novel, as shown by the challenges presented by functional annotation and taxonomic assignment. The DNA viral community is dominated by tailed bacteriophages belonging to *Siphoviridae*, *Podoviridae* and *Myoviridae* families. Members of single-strand DNA families such as *Microviridae* and *Inoviridae* were retrieved, as well as *Bicaudaviridae*. These families are characterised by small genomes and are underrepresented in sequence databases, which makes them more challenging to detect [93]. This lack of representation means that the sequences retrieved in this study are going to contribute to the knowledge expansion about environmental viruses which has been going on for well over a decade, with no sign of decrease yet [22].

Viral particles have a different structure than organisms, and lack a metabolism [94], whereas living beings can rely on homeostatic mechanisms to face changes in external factors such as pH changes and oxidative stress [95, 96]. Temperate viruses, furthermore, are induced and enter the lytic cycle as a reaction to some DNA-damaging stresses. These factors are effective when looking at the reactions of both viruses and prokaryotes to the different conditions that were applied in the experimental setup. Viruses show a clear dichotomy between organic overload and basic pH (Spearman’s *⍴* = − 0.69, *p* < 0.001), while prokaryotes show the greatest differences between basic pH and microaeration (Spearman’s *⍴* = − 0.40, *p* < 0.005).

This said, there are characteristic responses to conditions, as shown by the log ratio clustering (Fig. 3). Viruses show these trends very clearly, and these can be linked to the presence of specific genes in the clusters. For instance, cluster “3” comprises viral genomes which include the *yopX* gene. This gene is known to be involved in the regulation of the life cycle of temperate bacteriophages [79], thus suggesting that members of this group are temperate, and *yopX* is involved in their induction. Among the members of cluster “3” is *Siphoviridae* sp. 0142, one of the most abundant viral genomes hereby retrieved, which encodes this gene.

The relative abundance of viruses is little affected by the action of H_2_O_2_. The literature regarding the effect of H_2_O_2_ on bacteriophages is scarce and focuses on the effects of H_2_O_2_ vapour, but it appears that non-enveloped viruses, as tailed bacteriophages, are more resistant to oxidation than enveloped viruses. The same studies show that the presence of a complex medium, in this case cattle manure, is able to shield the viral particles from the effect of the peroxide, either by acting as a physical barrier or by reacting with the oxidative agent, thereby diminishing its concentration [97].

Microorganisms, as well, respond differently to atmospheric air and H_2_O_2_. Both conditions are supposed to put microorganisms in a state of oxidative stress; however, H_2_O_2_ is a much stronger oxidising agent and, as consequence, several species of *Bacteria* and *Archaea* are less able to face and survive the damage. Since *Archaea* are anaerobes and at best oxytolerant, they are heavily affected by strong oxidative stress (Additional file 3).

It can be striking to observe that archaeal species, which are so reactive to oxidative stresses, increase during organic overload. Organic overload is associated with an accumulation of VFAs and, consequently, the decrease of pH. This leads to inhibition of methanogenesis; however, this process takes several days to unfold, and the brief time span of this experiment could not allow it. Measurements of methane yield and VFA, particularly acetate concentration, are consistent with this explanation (Additional file 2, Fig. 1).

Some bacterial species decrease steadily in the presence of atmospheric air, the majority of which do not feature integrated proviruses. Accordingly, it can be speculated that bacteria more resistant to oxidative stress caused by microaeration are more likely to have integrated prophages. In fact, both *T. denitrificans* and the *Pseudomonas* genus are known as facultatively anaerobic [81, 98]: this clearly coincides with their ability to withstand a moderate oxidative stress as microaeration, but not a stronger one as the injection of H_2_O_2_. Both genomes have integrated phages, belonging to the *Myoviridae* and *Siphoviridae* families, respectively.

While 70% of the prokaryotic genes were annotated, this proportion drops to 30% in viral genomes, a proportion consistent with typical metaviromic studies [21, 69]. This striking discrepancy in terms of unclassified genes reiterates how vast the proportion of unknown genes in the viral world is. Yet, the annotated genes reveal the important role viruses play in shaping the microbial community of AD. Mobile Genetic Elements, viruses included, often contribute to the metabolic capability of their hosts by carrying genes conferring evolutionary advantage to the host [99], and the same can be observed in this community.

As mentioned before, viruses code for a considerable number of genes belonging to GT4 and GT2 families. These are enzymatic families of importance for biofilm synthesis, and it is known that in some bacterial species the lysogenic infection of a temperate phage increases the production of biofilm, benefitting both host and virus [100]. If such a phenomenon is confirmed to occur in AD environments, it will be reasonable to ponder the role of proviruses in the spreading of these genes.

The reductive acetyl-CoA pathway, also known as the Wood-Ljungdahl pathway, is a metabolic pathway characteristic of homoacetogenic bacteria and archaea. It allows the fixation of CO_2_ and synthesis of acetate, which is then used by acetoclastic archaea to produce methane. Other species, known as syntrophic acetate oxidising bacteria, employ the reverse Wood-Ljungdahl pathway to digest acetate into H_2_ and CO_2_, both consumed by hydrogenotrophic archaea. Biochemical evidences regarding the activity of the WL pathway have been reported for a limited number of isolated species: the species dubbed as “acetate-oxidising, rod-shaped bacterium” (AOR) [97], *Clostridium ultunense* [101], *Thermoacetogenium phaeum* [102], *Pseudothermotoga lettingae* [103], *Syntrophaceticus schinkii* [104] and *Tepidanaerobacter acetatoxydans* [105]. Genes belonging to these pathways were found in genomes retrieved in the present study, more specifically in 10 members of the *Firmicutes* phyla and one *Chloroflexi*. Members of both the *Firmicutes* and *Chloroflexi* phyla bacteria have been reported as either capable of converting acetate to CO_2_ through the reverse WL pathway or as potential syntrophic acetate oxidising bacteria based on gene annotations [87–89]. However, alternative WL pathways mediated by the glycine cleavage system and tetrahydrofolate pathway have been proposed in recent studies [2, 106, 107]. Some genes belonging to these pathways are potentially involved in bacteriophage-mediated HGT: *Siphoviridae* sp. 0243 is particularly noteworthy, as its genome includes five genes of the GSRP pathway. The presence of these genes can derive from previous excision. The integration of *Siphoviridae* sp. 0243 in another bacterial genome can, in theory, confer to the host new metabolic capabilities. However, phage-mediated HGT of these genes has never been previously reported and can be targeted in future studies. The genome of *Siphoviridae* sp. 0163, similarly, includes an enzyme of the CBM56 family, involved in the degradation of polysaccharides, and thus could confer this metabolic function to its host via HGT. As a last example, five free viruses and six proviruses code for proteins annotated with the Gene Ontology term GO:0006979, which groups genes mediating oxidative stress response, and thus might increase the host’s survivability to oxygen exposure. As a matter of fact, *Clostridiales* sp. 030 and *Synergistaceae* sp. 019, both of which include one of said proviruses, also show a positive log ratio under O_2_ exposure.

The results also reveal some of the adaptations these parasites use against their hosts.

It is known that, in bacteriophages parasitizing *E. coli*, tail spikes present sialidases which degrade the host’s coat of polysialic acid, allowing the interaction between phage and host [108]. Other depolymerases are known to enact similar processes in other bacteriophages [109]. However, our results indicate not only a presence of sialidases/neuraminidases, but an overrepresentation thereof, with respect to prokaryotic genomes. Hence, it is possible that these enzymes have an important, hitherto overlooked role in the mechanism of infection. In fact, 51% of the ORFs assigned to the GH33, GT2 and GT4 families (47 out of 91) are annotated by eggNOG as tail proteins or tape measure proteins, supporting the idea that these enzymatic activities are especially relevant in phage/host interactions.

Two of the archaeal genomes retrieved in this study, *M. mazei* sp. 049 and *M. flavescens* sp. 114, incorporate in their sequence integrated proviruses of the Siphoviridae family. While most *Siphoviridae* are bacteriophages, there’s evidence that some members of the family infect *Archaea*, including the methanogenic species *Methanoculleus bourgensis* and *Methanobacterium formicicum* [110, 111]. These members of *Siphoviridae* are lytic, i.e., do not integrate in the host of the genome; the newly recovered genomes show the existence of lysogenic archaeal *Siphoviridae* as well.

In order to better understand the dynamics between viruses and hosts, the reads from a large number of AD experiments were mapped on integrated proviruses and host genomes retrieved in this analysis. These experiments widened the exploration of prophage behaviour, allowing the identification of specific environmental conditions favouring prophage induction. Additionally, they provide insights on the presence/absence of each integrated prophage in the MAG across different conditions. The first observation is that, when host and virus are not both present, usually the virus is missing from the experiment. This is consistent with the idea that viruses tend to co-exist with their hosts, and that different communities may consist of different viral species even in the case where the same host is present.

Simplified medium communities are characterised by high viral abundance, as well as low number of reads mapped on the genomes retrieved in the current experiment. These characteristics are easily explained by the growth conditions: the specific nutrient source imposes a strong selection on the microbial species, e.g. the hydrolytic guild in the case of acetate-based media. It is feasible to think that such stresses lead to the induction of integrated proviruses. However, possible biases should be taken into account. A small number of reads mapping both on the host and the provirus may skew the ratio due to stochasticity; another factor which can lead to a high virus/host coverage ratio is the presence of alternative hosts.

## Conclusions

The shift to a circular economy and the reduction of greenhouse gas emissions is pressing and requires a massive effort in terms of technology adoption. In this context, AD is a widely used technology; nevertheless, a key component of the process, the virome, is still relatively unknown. To our knowledge, this is the first time in which the viral community of the AD was inspected under a great variety of different conditions. This study reveals the pervasiveness of viruses in the AD microbiome. The data retrieved in this work and the analyses hereby carried out lay the bases towards the understanding of the complex role of the viral community in AD.

Viral genomes featuring genes of relevance in the AD process were retrieved, opening up the possibility that HGT is carried out by viruses. Shocks impacted viruses and microbes in different ways, highlighting four taxonomically heterogeneous clusters of species.

Broadening the analysis to a wide array of AD studies enabled the consideration of the effect of more environmental parameters, such as temperature and medium composition, on the abundance of temperate viruses and their hosts. It also reveals that the viral community is more mutable than the microbial one, as viruses are often not found despite the presence of their hosts, while the opposite is much rarer. More in-depth studies on the microbiomes of samples of the AD database might elucidate if and how metabolic stresses and starvation placed on some microorganisms by the simplified feedstock affect phage induction. Although this study is limited to the analysis of DNA viruses, it can be expanded in the future to include the RNA viral community. In the next future, knowledge about the interactions between viruses and their host will have the potential to improve the efficiency of the AD process and the production of biogas, as it is already done in different environments such as wastewater treatment plants, food surfaces and even the human body. More studies with innovative approaches are needed to understand thoroughly the effects of conditions typical of AD on the lifestyle of the viruses that inhabit this engineered ecosystem. On a shorter timescale, the newly discovered viral genomes contribute to the ever-growing diversity of environmental viruses which is shifting our understanding of these entities.

## Supplementary Information

**Additional file 1:** Supplementary methods regarding details of experimental setup, bin curation, calculation of relative abundance and taxonomy assignment. **Figure 1.** UpSet plot of viral sequences predicted by different tools. **Figure 2.** Scatter plots comparing CPM and CheckM relative abundance values. **Figure 3.** Relative abundance expressed in count per million (CPM) of the most abundant prokaryotic and viral genomes **Figure 4.** Heatmaps displaying variations of phages and MAGs compared to supernatant and pellet control. **Figure 5.** Distribution of the provirus/host CPM abundance ratios across all datasets.

**Additional file 2: Table 1.** Measurements of VFA and methane yield.

**Additional file 3: Table 2.** General information about the prokaryotic and viral MAGs retrieved in this study: genome length, completeness, contamination, taxonomy, relative abundances, log ratios with respect to the average.

**Additional file 4: Table 3.** Functional annotation of MAGs with eggNOG mapper, KEMET, dbCAN, gutSMASH.

**Additional file 5: Table 4.** Coverage of prophage-host couples in samples from the AD database, results of hierarchical clustering of the samples, linear regressions between the relative abundances of proviruses and hosts.

## Data Availability

Raw reads data are available at NCBI SRA ( https://www.ncbi.nlm.nih.gov/sra) under the BioProject ID PRJNA767833.

## Abbreviations

AD: Anaerobic digestion
WWTP: Wastewater treatment plants
HGT: Horizontal gene transfer
VFA: Volatile fatty acids
TCD: Thermal conductivity detector
FID: Flame ionisation detector
MAG: Metagenome-assembled genome
ORF: Open reading frame
CPM: Count per million
pVOG: Prokaryotic Virus Orthologous Group
KEGG: Kyoto Encyclopedia of Genes and Genomes
WLP: Wood-Ljungdahl pathway
GSRP: Glycine synthase-reductase pathway
RGP: Reductive glycine pathway

## References

[1] Dutta S, He M, Xiong X, Tsang DCW (2021). Sustainable management and recycling of food waste anaerobic digestate: a review. Bioresour Technol.

[2] Campanaro S, Treu L, Rodriguez-R LM, Kovalovszki A, Ziels RM, Maus I (2020). New insights from the biogas microbiome by comprehensive genome-resolved metagenomics of nearly 1600 species originating from multiple anaerobic digesters. Biotechnol Biofuels.

[3] Ma S, Jiang F, Huang Y, Zhang Y, Wang S, Fan H (2021). A microbial gene catalog of anaerobic digestion from full-scale biogas plants. GigaScience..

[4] Carabeo-Pérez A, Guerra-Rivera G, Ramos-Leal M, Jiménez-Hernández J (2019). Metagenomic approaches: effective tools for monitoring the structure and functionality of microbiomes in anaerobic digestion systems. Appl Microbiol Biotechnol.

[5] Paez-Espino D, Eloe-Fadrosh EA, Pavlopoulos GA, Thomas AD, Huntemann M, Mikhailova N (2016). Uncovering Earth’s virome. Nature..

[6] Wommack KE, Colwell RR (2000). Virioplankton: viruses in aquatic ecosystems. Microbiol Mol Biol Rev.

[7] Wu Q, Liu W-T (2009). Determination of virus abundance, diversity and distribution in a municipal wastewater treatment plant. Water Res.

[8] Shapiro OH, Kushmaro A, Brenner A (2010). Bacteriophage predation regulates microbial abundance and diversity in a full-scale bioreactor treating industrial wastewater. ISME J.

[9] Calusinska M, Marynowska M, Goux X, Lentzen E, Delfosse P (2016). Analysis of ds DNA and RNA viromes in methanogenic digesters reveals novel viral genetic diversity. Environ Microbiol.

[10] Willenbücher K, Wibberg D, Huang L, Conrady M, Ramm P, Gätcke J (2022). Phage genome diversity in a biogas-producing microbiome analyzed by Illumina and Nanopore GridION sequencing. Microorganisms..

[11] Heyer R, Schallert K, Siewert C, Kohrs F, Greve J, Maus I (2019). Metaproteome analysis reveals that syntrophy, competition, and phage-host interaction shape microbial communities in biogas plants. Microbiome..

[12] Zhang J, Gao Q, Zhang Q, Wang T, Yue H, Wu L (2017). Bacteriophage–prokaryote dynamics and interaction within anaerobic digestion processes across time and space. Microbiome..

[13] Nanda AM, Thormann K, Frunzke J (2015). Impact of spontaneous prophage induction on the fitness of bacterial populations and host-microbe interactions. Margolin W, editor. J Bacteriol.

[14] Choi J, Kotay SM, Goel R (2010). Various physico-chemical stress factors cause prophage induction in *Nitrosospira multiformis* 25196- an ammonia oxidizing bacteria. Water Res..

[15] Brüssow H, Bruttin A, Desiere F, Lucchini S, Foley S (1998). Molecular ecology and evolution of *Streptococcus thermophilus* bacteriophages–a review. Virus Genes..

[16] Pan D, Watson R, Wang D, Tan ZH, Snow DD, Weber KA (2014). Correlation between viral production and carbon mineralization under nitrate-reducing conditions in aquifer sediment. ISME J.

[17] Brussaard CPD (2004). Viral control of phytoplankton populations--a review. J Eukaryot Microbiol.

[18] Suttle CA (2007). Marine viruses — major players in the global ecosystem. Nat Rev Microbiol.

[19] Harrison E, Brockhurst MA (2017). Ecological and evolutionary benefits of temperate phage: what does or doesn’t kill you makes you stronger. BioEssays..

[20] Krishnamurthy SR, Wang D (2017). Origins and challenges of viral dark matter. Virus Res.

[21] Camarillo-Guerrero LF, Almeida A, Rangel-Pineros G, Finn RD, Lawley TD (2021). Massive expansion of human gut bacteriophage diversity. Cell..

[22] Roux S, Páez-Espino D, Chen I-MA, Palaniappan K, Ratner A, Chu K (2021). IMG/VR v3: an integrated ecological and evolutionary framework for interrogating genomes of uncultivated viruses. Nucleic Acids Res.

[23] Kieft K, Zhou Z, Anantharaman K (2020). VIBRANT: automated recovery, annotation and curation of microbial viruses, and evaluation of viral community function from genomic sequences. Microbiome..

[24] Nayfach S, Camargo AP, Schulz F, Eloe-Fadrosh E, Roux S, Kyrpides NC (2021). CheckV assesses the quality and completeness of metagenome-assembled viral genomes. Nat Biotechnol.

[25] Ren J, Ahlgren NA, Lu YY, Fuhrman JA, Sun F (2017). VirFinder: a novel k-mer based tool for identifying viral sequences from assembled metagenomic data. Microbiome..

[26] Guo J, Bolduc B, Zayed AA, Varsani A, Dominguez-Huerta G, Delmont TO (2021). VirSorter2: a multi-classifier, expert-guided approach to detect diverse DNA and RNA viruses. Microbiome..

[27] Arndt D, Grant JR, Marcu A, Sajed T, Pon A, Liang Y (2016). PHASTER: a better, faster version of the PHAST phage search tool. Nucleic Acids Res.

[28] Fang Z, Tan J, Wu S, Li M, Xu C, Xie Z (2019). PPR-Meta: a tool for identifying phages and plasmids from metagenomic fragments using deep learning. GigaScience..

[29] Dutilh BE, Cassman N, McNair K, Sanchez SE, Silva GGZ, Boling L (2014). A highly abundant bacteriophage discovered in the unknown sequences of human faecal metagenomes. Nat Commun.

[30] Rossi A, Treu L, Toppo S, Zschach H, Campanaro S, Dutilh BE (2020). Evolutionary study of the crassphage virus at gene level. Viruses..

[31] Yutin N, Benler S, Shmakov SA, Wolf YI, Tolstoy I, Rayko M (2021). Analysis of metagenome-assembled viral genomes from the human gut reveals diverse putative CrAss-like phages with unique genomic features. Nat Commun.

[32] Simmonds P, Adams MJ, Benkő M, Breitbart M, Brister JR, Carstens EB (2017). Virus taxonomy in the age of metagenomics. Nat Rev Microbiol.

[33] Batinovic W, Knowler R, Stanton R (2019). Bacteriophages in natural and artificial environments. Pathogens..

[34] Cristobal-Cueto P, García-Quintanilla A, Esteban J, García-Quintanilla M (2021). Phages in food industry biocontrol and bioremediation. Antibiotics..

[35] Jassim SAA, Limoges RG, El-Cheikh H (2016). Bacteriophage biocontrol in wastewater treatment. World J Microbiol Biotechnol.

[36] Kotay SM, Datta T, Choi J, Goel R (2011). Biocontrol of biomass bulking caused by *Haliscomenobacter hydrossis* using a newly isolated lytic bacteriophage. Water Res..

[37] Rodriguez-R LM, Gunturu S, Harvey WT, Rosselló-Mora R, Tiedje JM, Cole JR (2018). The Microbial Genomes Atlas (MiGA) webserver: taxonomic and gene diversity analysis of Archaea and Bacteria at the whole genome level. Nucleic Acids Res.

[38] Ghosh D, Roy K, Williamson KE, White DC, Wommack KE, Sublette KL (2008). Prevalence of lysogeny among soil bacteria and presence of 16S rRNA and *trzN* genes in viral-community DNA. Appl Environ Microbiol.

[39] Mahuku GS (2004). A simple extraction method suitable for PCR-based analysis of plant, fungal, and bacterial DNA. Plant Mol Biol Report.

[40] Santos-Medellin C, Zinke LA, ter Horst AM, Gelardi DL, Parikh SJ, Emerson JB (2021). Viromes outperform total metagenomes in revealing the spatiotemporal patterns of agricultural soil viral communities. ISME J.

[41] Bolger AM, Lohse M, Usadel B (2014). Trimmomatic: a flexible trimmer for Illumina sequence data. Bioinformatics..

[42] Li D, Liu C-M, Luo R, Sadakane K, Lam T-W (2015). MEGAHIT: an ultra-fast single-node solution for large and complex metagenomics assembly via succinct de Bruijn graph. Bioinformatics..

[43] Gurevich A, Saveliev V, Vyahhi N, Tesler G (2013). QUAST: quality assessment tool for genome assemblies. Bioinformatics..

[44] Langmead B, Salzberg SL (2012). Fast gapped-read alignment with Bowtie 2. Nat Methods.

[45] Kang DD, Li F, Kirton E, Thomas A, Egan R, An H (2019). MetaBAT 2: an adaptive binning algorithm for robust and efficient genome reconstruction from metagenome assemblies. PeerJ..

[46] Kang DD, Froula J, Egan R, Wang Z (2015). MetaBAT, an efficient tool for accurately reconstructing single genomes from complex microbial communities. PeerJ..

[47] Parks DH, Imelfort M, Skennerton CT, Hugenholtz P, Tyson GW (2015). CheckM: assessing the quality of microbial genomes recovered from isolates, single cells, and metagenomes. Genome Res.

[48] Virtanen P, Gommers R, Oliphant TE, Haberland M, Reddy T, Cournapeau D (2020). SciPy 1.0: fundamental algorithms for scientific computing in Python. Nat Methods.

[49] Friedman J, Alm EJ. Inferring correlation networks from genomic survey data. 2012. 10.1371/journal.pcbi.1002687. 10.1371/journal.pcbi.1002687 PMC3447976 23028285

[50] Chaumeil PA, Mussig AJ, Hugenholtz P, Parks DH. GTDB-Tk: a toolkit to classify genomes with the Genome Taxonomy Database. 2020. 10.1093/bioinformatics/btz848. 10.1093/bioinformatics/btz848 PMC7703759 31730192

[51] Eddy SR (1998). Profile hidden Markov models. Bioinformatics..

[52] Grazziotin AL, Koonin EV, Kristensen DM (2017). Prokaryotic Virus Orthologous Groups (pVOGs): a resource for comparative genomics and protein family annotation. Nucleic Acids Res.

[53] Hyatt D, Chen G-L, LoCascio PF, Land ML, Larimer FW, Hauser LJ (2010). Prodigal: prokaryotic gene recognition and translation initiation site identification. BMC Bioinformatics.

[54] Borges AL, Lou YC, Sachdeva R, Al-Shayeb B, Jaffe AL, Lei S, et al. Stop codon recoding is widespread in diverse phage lineages and has the potential to regulate translation of late stage and lytic genes. bioRxiv. 2021. 10.1101/2021.08.26.457843.

[55] Huerta-Cepas J, Forslund K, Coelho LP, Szklarczyk D, Jensen LJ, von Mering C (2017). Fast genome-wide functional annotation through orthology assignment by eggNOG-mapper. Mol Biol Evol.

[56] Palù M, Basile A, Zampieri G, Treu L, Rossi A, Morlino MS (2022). KEMET–A python tool for KEGG Module evaluation and microbial genome annotation expansion. Comput Struct Biotechnol J..

[57] Yin Y, Mao X, Yang J, Chen X, Mao F, Xu Y (2012). dbCAN: a web resource for automated carbohydrate-active enzyme annotation. Nucleic Acids Res.

[58] Pascal Andreu V, Roel-Touris J, Dodd D, Fischbach MA, Medema MH (2021). The gutSMASH web server: automated identification of primary metabolic gene clusters from the gut microbiota. Nucleic Acids Res.

[59] Binnenkade L, Teichmann L, Thormann KM. Iron triggers λSo prophage induction and release of extracellular DNA in *Shewanella oneidensis* MR-1 Biofilms. Spormann AM, editor. Appl Environ Microbiol. 2014;80:5304–16. 10.1128/AEM.01480-14 PMC4136115 24951794

[60] Long A, McDaniel LD, Mobberley J, Paul JH (2008). Comparison of lysogeny (prophage induction) in heterotrophic bacterial and *Synechococcus* populations in the Gulf of Mexico and Mississippi river plume. ISME J..

[61] Harris SM, Yue W-F, Olsen SA, Hu J, Means WJ, McCormick RJ (2012). Salt at concentrations relevant to meat processing enhances Shiga toxin 2 production in *Escherichia coli* O157:H7. Int J Food Microbiol..

[62] Boe K, Batstone DJ, Steyer J-P, Angelidaki I (2010). State indicators for monitoring the anaerobic digestion process. Water Res.

[63] Tsapekos P, Kougias PG, Vasileiou SA, Lyberatos G, Angelidaki I (2017). Effect of micro-aeration and inoculum type on the biodegradation of lignocellulosic substrate. Bioresour Technol.

[64] Angelidaki I, Treu L, Tsapekos P, Luo G, Campanaro S, Wenzel H (2018). Biogas upgrading and utilization: current status and perspectives. Biotechnol Adv.

[65] Liu J, Jia R, Wang Y, Wei Y, Zhang J, Wang R (2017). Does residual H_2_O_2_ result in inhibitory effect on enhanced anaerobic digestion of sludge pretreated by microwave-H_2_O_2_ pretreatment process?. Environ Sci Pollut Res..

[66] Touchon M, Bernheim A, Rocha EP (2016). Genetic and life-history traits associated with the distribution of prophages in bacteria. ISME J.

[67] Yuan Y, Gao M (2017). Jumbo Bacteriophages: An Overview. Front Microbiol.

[68] Roux S, Adriaenssens EM, Dutilh BE, Koonin EV, Kropinski AM, Krupovic M (2019). Minimum information about an uncultivated virus genome (MIUViG). Nat Biotechnol.

[69] Nayfach S, Páez-Espino D, Call L, Low SJ, Sberro H, Ivanova NN (2021). Metagenomic compendium of 189,680 DNA viruses from the human gut microbiome. Nat Microbiol.

[70] Fontana A, Campanaro S, Treu L, Kougias PG, Cappa F, Morelli L (2018). Performance and genome-centric metagenomics of thermophilic single and two-stage anaerobic digesters treating cheese wastes. Water Res.

[71] Kakuk B, Wirth R, Maróti G, Szuhaj M, Rakhely G, Laczi K (2021). Early response of methanogenic archaea to H_2_ as evaluated by metagenomics and metatranscriptomics. Microb Cell Factories..

[72] Tian H, Fotidis IA, Kissas K, Angelidaki I (2018). Effect of different ammonia sources on aceticlastic and hydrogenotrophic methanogens. Bioresour Technol.

[73] Maus I, Wibberg D, Stantscheff R, Eikmeyer F-G, Seffner A, Boelter J (2012). Complete genome sequence of the hydrogenotrophic, methanogenic archaeon *Methanoculleus bourgensis* strain MS2(T), Isolated from a sewage sludge digester. J Bacteriol..

[74] Evans PN, Boyd JA, Leu AO, Woodcroft BJ, Parks DH, Hugenholtz P (2019). An evolving view of methane metabolism in the Archaea. Nat Rev Microbiol.

[75] Ziels RM, Sousa DZ, Stensel HD, Beck DAC (2018). DNA-SIP based genome-centric metagenomics identifies key long-chain fatty acid-degrading populations in anaerobic digesters with different feeding frequencies. ISME J.

[76] Calusinska M, Goux X, Fossépré M, Muller EEL, Wilmes P, Delfosse P (2018). A year of monitoring 20 mesophilic full-scale bioreactors reveals the existence of stable but different core microbiomes in bio-waste and wastewater anaerobic digestion systems. Biotechnol Biofuels.

[77] Lucas R, Kuchenbuch A, Fetzer I, Harms H, Kleinsteuber S. Long-term monitoring reveals stable and remarkably similar microbial communities in parallel full-scale biogas reactors digesting energy crops. FEMS Microbiol Ecol. 2015;91 Available from: https://academic.oup.com/femsec/article-lookup/doi/10.1093/femsec/fiv004. Cited 2021 Oct 15. 10.1093/femsec/fiv004 25764564

[78] Sun L, Liu T, Müller B, Schnürer A (2016). The microbial community structure in industrial biogas plants influences the degradation rate of straw and cellulose in batch tests. Biotechnol Biofuels.

[79] Yasmin A, Kenny JG, Shankar J, Darby AC, Hall N, Edwards C (2010). Comparative genomics and transduction potential of *Enterococcus faecalis* temperate bacteriophages. J Bacteriol.

[80] Ahring BK, Sandberg M, Angelidaki I (1995). Volatile fatty acids as indicators of process imbalance in anaerobic digestors. Appl Microbiol Biotechnol.

[81] Tan W-B, Jiang Z, Chen C, Yuan Y, Gao L-F, Wang H-F, et al. *Thiopseudomonas denitrificans* gen. nov., sp. nov., isolated from anaerobic activated sludge. Int J Syst Evol Microbiol. 2015;65:225–9. 10.1099/ijs.0.064634-0 25326445

[82] Campanaro S, Treu L, Kougias PG, Luo G, Angelidaki I (2018). Metagenomic binning reveals the functional roles of core abundant microorganisms in twelve full-scale biogas plants. Water Res.

[83] Cavaliere M, Feng S, Soyer OS, Jiménez JI (2017). Cooperation in microbial communities and their biotechnological applications. Environ Microbiol.

[84] Rankin DJ, Rocha EPC, Brown SP (2011). What traits are carried on mobile genetic elements, and why?. Heredity..

[85] Sundberg C, Al-Soud WA, Larsson M, Alm E, Yekta SS, Svensson BH (2013). 454 pyrosequencing analyses of bacterial and archaeal richness in 21 full-scale biogas digesters. FEMS Microbiol Ecol.

[86] Hehemann J-H, Correc G, Barbeyron T, Helbert W, Czjzek M, Michel G (2010). Transfer of carbohydrate-active enzymes from marine bacteria to Japanese gut microbiota. Nature..

[87] Buhlmann CH, Mickan BS, Jenkins SN, Tait S, Kahandawala TKA, Bahri PA (2019). Ammonia stress on a resilient mesophilic anaerobic inoculum: methane production, microbial community, and putative metabolic pathways. Bioresour Technol.

[88] Mosbæk F, Kjeldal H, Mulat DG, Albertsen M, Ward AJ, Feilberg A (2016). Identification of syntrophic acetate-oxidizing bacteria in anaerobic digesters by combined protein-based stable isotope probing and metagenomics. ISME J.

[89] Ruiz-Sánchez J, Campanaro S, Guivernau M, Fernández B, Prenafeta-Boldú FX (2018). Effect of ammonia on the active microbiome and metagenome from stable full-scale digesters. Bioresour Technol.

[90] Chen S, Zamudio Cañas EM, Zhang Y, Zhu Z, He Q (2012). Impact of substrate overloading on archaeal populations in anaerobic digestion of animal waste. J Appl Microbiol.

[91] Kalamaras SD, Vasileiadis S, Karas P, Angelidaki I, Kotsopoulos TA (2020). Microbial adaptation to high ammonia concentrations during anaerobic digestion of manure-based feedstock: biomethanation and 16S rRNA gene sequencing. J Chem Technol Biotechnol.

[92] de Jonge PA, Nobrega FL, Brouns SJJ, Dutilh BE (2019). Molecular and evolutionary determinants of bacteriophage host range. Trends Microbiol.

[93] Nasir A, Forterre P, Kim KM, Caetano-Anollés G (2014). The distribution and impact of viral lineages in domains of life. Front Microbiol.

[94] Aljabali AA, Hassan SS, Pabari RM, Shahcheraghi SH, Mishra V, Charbe NB (2021). The viral capsid as novel nanomaterials for drug delivery. Future Sci OA.

[95] Slonczewski JL, Fujisawa M, Dopson M, Krulwich TA (2009). Cytoplasmic pH measurement and homeostasis in bacteria and archaea. Adv Microb Physiol.

[96] Khan MZ, Singha B, Ali MF, Taunk K, Rapole S, Gourinath S, et al. Redox homeostasis in *Mycobacterium tuberculosis* is modulated by a novel actinomycete-specific transcription factor. EMBO J. 2021;40:e106111. 10.15252/embj.2020106111 PMC8280819 34018220

[97] Wood JP, Richter W, Sunderman M, Calfee MW, Serre S, Mickelsen L (2020). Evaluating the environmental persistence and inactivation of MS2 bacteriophage and the presumed Ebola virus surrogate phi6 using low concentration hydrogen peroxide vapor. Environ Sci Technol.

[98] Yokoyama K, Yumura M, Honda T, Ajitomi E (2016). Characterization of denitrification and net N _2_ O-reduction properties of novel aerobically N _2_ O-reducing bacteria. Soil Sci Plant Nutr.

[99] Johnson CN, Sheriff EK, Duerkop BA, Chatterjee A (2021). Let Me Upgrade You: impact of mobile genetic elements on enterococcal adaptation and evolution. Margolin W, editor. J Bacteriol.

[100] Tan D, Hansen MF, de Carvalho LN, Røder HL, Burmølle M, Middelboe M (2020). High cell densities favor lysogeny: induction of an H_2_0 prophage is repressed by quorum sensing and enhances biofilm formation in *Vibrio anguillarum*. ISME J..

[101] Schnurer A, Schink B, Svensson BH. *Clostridium ultunense* sp. nov., a mesophilic bacterium oxidizing acetate in syntrophic association with a hydrogenotrophic methanogenic bacterium. Int J Syst Bacteriol. 1996;46:1145–52. 10.1099/00207713-46-4-1145 8863449

[102] Hattori S, Kamagata Y, Hanada S, Shoun H. *Thermacetogenium phaeum* gen. nov., sp. nov., a strictly anaerobic, thermophilic, syntrophic acetate-oxidizing bacterium. Int J Syst Evol Microbiol. 2000;50:1601–9. 10.1099/00207713-50-4-1601 10939667

[103] Balk M, Weijma J, Stams AJM. *Thermotoga lettingae* sp. nov., a novel thermophilic, methanol-degrading bacterium isolated from a thermophilic anaerobic reactor. Int J Syst Evol Microbiol. 2002;52:1361–8. 10.1099/00207713-52-4-1361 12148651

[104] Westerholm M, Roos S, Schnürer A. *Syntrophaceticus schinkii* gen. nov., sp. nov., an anaerobic, syntrophic acetate-oxidizing bacterium isolated from a mesophilic anaerobic filter. FEMS Microbiol Lett. 2010;309(1):100–4. 10.1111/j.1574-6968.2010.02023.x. 10.1111/j.1574-6968.2010.02023.x 20546311

[105] Westerholm M, Roos S, Schnürer A. *Tepidanaerobacter acetatoxydans* sp. nov., an anaerobic, syntrophic acetate-oxidizing bacterium isolated from two ammonium-enriched mesophilic methanogenic processes. Syst Appl Microbiol. 2011;34:260–6. 10.1016/j.syapm.2010.11.018 21498020

[106] Nobu MK, Narihiro T, Rinke C, Kamagata Y, Tringe SG, Woyke T (2015). Microbial dark matter ecogenomics reveals complex synergistic networks in a methanogenic bioreactor. ISME J.

[107] Zhu X, Campanaro S, Treu L, Seshadri R, Ivanova N, Kougias PG (2020). Metabolic dependencies govern microbial syntrophies during methanogenesis in an anaerobic digestion ecosystem. Microbiome..

[108] Bull JJ, Vimr ER, Molineux IJ (2010). A tale of tails: Sialidase is key to success in a model of phage therapy against K1-capsulated *Escherichia coli*. Virology..

[109] Pires DP, Oliveira H, Melo LDR, Sillankorva S, Azeredo J (2016). Bacteriophage-encoded depolymerases: their diversity and biotechnological applications. Appl Microbiol Biotechnol.

[110] Wolf S, Fischer MA, Kupczok A, Reetz J, Kern T, Schmitz RA (2019). Characterization of the lytic archaeal virus Drs3 infecting *Methanobacterium formicicum*. Arch Virol..

[111] Weidenbach K, Wolf S, Kupczok A, Kern T, Fischer MA, Reetz J (2021). Characterization of Blf4, an archaeal lytic virus targeting a member of the methanomicrobiales. Viruses..

