# Supplementary material for: Analysis of the anaerobic digestion metagenome under environmental stresses stimulating prophage induction
Source: Microbiome. 2022 Aug 15;10:125. doi: 10.1186/s40168-022-01316-w (PMC9377139; doi:10.1186/s40168-022-01316-w)
Supplement: Supplementary file 2 — Additional file 1: Supplementary methods regarding details of experimental setup, bin curation, calculation of relative abundance and taxonomy assignment. Figure 1. UpSet plot of viral sequences predicted by different tools. Figure 2. Scatter plots comparing CPM and CheckM relative abundance values. Figure 3. Relative abundance expressed in count per million (CPM) of the most abundant prokaryotic and viral genomes Figure 4. Heatmaps displaying variations of phages and MAGs compared to supernatant and pellet control. Figure 5. Distribution of the provirus/host CPM abundance ratios across all datasets. [file 40168_2022_1316_MOESM2_ESM.docx]

Analysis of the anaerobic digestion metagenome under environmental stresses stimulating prophage induction

Alessandro Rossi^1a^, Maria Silvia Morlino^1a^, Maria Gaspari^2^, Arianna Basile^1^, Panagiotis Kougias^3^, Laura Treu^1^*, Stefano Campanaro^1,4^

**^1^** Department of Biology, University of Padua, via U. Bassi 58/b, 35131 Padova, Italy.

**^2^** Department of Hydraulics, Soil Science and Agricultural Engineering, Faculty of Agriculture, Aristotle University of Thessaloniki, GR-54124 Thessaloniki, Greece.

**^3^** Soil and Water Resources Institute, Hellenic Agricultural Organisation Demeter, Thermi, Thessaloniki 57001, Greece.

**^4^** CRIBI biotechnology center, University of Padua, via U. Bassi 58/b, 35131 Padova, Italy.

^a^ Equal contribution.

* Correspondence: laura.treu@unipd.it

**Batch assays experimental setup**

300 mL bottles were set up with 48 mL of inoculum from a continuous stirred tank reactor (CSTR) and 2 mL of cattle manure as feedstock, for a total volume of 50 mL. Organic overload was achieved by increasing the feedstock concentration. Three different concentrations were tested: 4, 6, and 8 g VS/L. Oxidation stress was performed by injecting atmospheric air in the bottles at 5, 10, and 15 mL O_2_/g VS, corresponding to 23.85, 47.7 and 71.55 mL of atmospheric air, respectively. Stronger oxidation was also achieved by supplementing bottles with H_2_O_2_ at a final concentration of 3 mM. Assays on the effect of pH were carried out by maintaining three sets of bottles at pH 5, 7, and 8.5. Adjustment of pH was made by injecting filter-sterilized solutions of 1 M NaOH and 1 M HCl. Two sets of bottles were treated with 25 and 75 μg mitomycin, respectively. Temperature stresses were carried out by keeping bottles in incubators at 45°C, 55°C, and 60°C, for the whole 24 hours of the experiment. Two assays were set up to test for osmotic stresses by supplementing the bottles with NaCl at the final concentrations of 1% (w/w) and 2% (w/w). Finally, four assays were set up with combinations of stresses: temperature 45°C x NaCl 1%; temperature 45°C x H_2_O_2_ 3 mM; temperature 55°C x NaCl 1%; temperature 55°C x H_2_O_2_ 3 mM.

**Read preprocessing and assembly**

Reads were filtered with Trimmomatic v0.39 [[1]](https://www.zotero.org/google-docs/?HFwM5h) with the parameters LEADING:20 TRAILING:20 SLIDINGWINDOW:4:20 MINLEN:70 and cleaned with BBDuk v38.86 using the parameters k=21, ktrim=r, mink=11, hdist=2 and using the “adapters'' and “phix” reference files. The reads of all pellet and supernatant samples were co-assembled using MEGAHIT v1.2.9 [[2]](https://www.zotero.org/google-docs/?kPdv1d) with a minimum contig length of 1000 and the meta-sensitive preset option. The quality of the co-assembly was assessed with QUAST v5.0.2 [[3]](https://www.zotero.org/google-docs/?pTY5xj). The filtered and cleaned reads were then mapped on the assembly with Bowtie 2 v2.3.5.1.

**Viral identification tools**

The assembled contigs were analysed with PPR-Meta v1.1, CheckV v0.7.0, VIBRANT v1.2.0 and the PHASTER web server [[4–7]](https://www.zotero.org/google-docs/?nvKObt) with default parameters. CheckV results were filtered excluding predictions with “not determined” quality and no viral genes detected. PPR-Meta predictions were filtered for viral scores of 0.75 or higher. VIBRANT and PHASTER predictions were carried forward with no pre-filtering. A first list of contigs classified as viral was defined by considering predictions made by either PHASTER alone, or at least two of the other programs. For integrated regions (identified specifically by PHASTER, VIBRANT and CheckV), the coordinates of start and end were taken from the output of PHASTER. If a PHASTER prediction was not available, then the coordinates were taken from the VIBRANT prediction. If a VIBRANT prediction was not available either, then CheckV coordinates were used. The set of viral predictions obtained was compared with the results of VirSorter2 (minimum viral score 0.5) and VirFinder (maximum p-value 0.05) [[8,9]](https://www.zotero.org/google-docs/?hmdxcj). Predictions in common between the two tools and the set of predictions used in this work were displayed with an UpSet plot (**Additional figure 1**).

**Binning and refinement**

A binning procedure was performed with MetaBAT2 v2.12.1 [[10,11]](https://www.zotero.org/google-docs/?cCN4xS) using a minimum bin size of 10,000 bp. Here, we refer to the output of the binning algorithm as “bins”, and to bins which have passed quality control and are thus considered representative of prokaryotic genomes as “MAGs”. The bins yielded by MetaBAT2 were evaluated and divided into Metagenome-Assembled Genomes (MAGs), viral MAGs and unclassified contigs. Bin quality, completeness and contamination were measured with CheckM v1.1.2 [[12]](https://www.zotero.org/google-docs/?WoRLM3) using the lineage_wf workflow and the qa command. Bins with CheckM completeness >=50% and contamination <=10% were deemed MAGs, and viral contigs belonging to them were considered as integrated viruses. When completeness and contamination in a bin did not pass the established CheckM thresholds for medium/high quality (completeness <50%; contamination >10%), only viral contigs in that bin were kept and considered together as a viral MAG, while the rest of the contigs was considered as unclassified. If the length of the viral MAG exceeded 200,000 bp, the bin was regarded as spurious under the assumption that the large majority of phage genomes has considerably lower dimensions, and each viral contig larger than 5,000 bp in these spurious bins was considered as an individual viral genome. Among contigs left unbinned, each viral prediction over 5,000 bp of length was considered as an individual viral genome.

Viral genomes resulting from this procedure whose length was shorter than 5,000 bp were not considered for the calculation of relative abundance; however, open reading frame (ORF) prediction, functional annotation and tentative taxonomic assignment were performed.

Finally, in order to assess whether the binning was effective in aggregating viral contigs together, CheckV was run again on the viral MAGs. When these consisted of more than one contig, the components were concatenated in arbitrary order.

**Relative abundance**

Relative abundance of prokaryotic and viral MAGs was calculated by performing count per million (CPM) normalisation on read counts obtained from the re-mapping of reads on the assembly. Raw read counts for each contig in each sample were obtained from the bam files with samtools v1.11 [[13]](https://www.zotero.org/google-docs/?thoYnw), with the coverage command, setting the minimum alignment quality score (*-q* option) to 20. Read counts were summed according to the grouping of the contigs into MAGs, yielding a raw read count for each putative genome. These read counts were then normalised for genome size and sequencing depth by calculating the CPM metric. For comparison, relative abundances were also calculated with CheckM software. The “coverage” module was used to process the BAM files generated by aligning the raw reads to the assembly. Then, the “profile” module was launched on the output to determine the results for each MAG and virMAG. For each sample, the column “%_binned_population” was compared to the respective CPM values by calculating the Spearman correlation coefficient. The statistical significance of the coefficient was evaluated as implemented in the python package SciPy v1.3.1 [[14]](https://www.zotero.org/google-docs/?YQcEQc). Furthermore, CPM and CheckM values were visualised in scatter plots (**Additional figure 2**).

The effect of the different shocks was evaluated by calculating the log ratio between the abundance of genomes in each shock and the mean abundance across all the conditions considered. In this context, a positive log ratio means an abundance higher than average and vice versa.

Euclidean distance was calculated from the log ratios, and the MAGs were clustered with an average linkage method. Clusters of genomes affected by similar shocks were evaluated separately for the 50 most abundant MAGs and viruses.

**Taxonomic assignment and functional annotation**

Prokaryotic MAGs were taxonomically assigned using GTDB-Tk v1.4.1 [[15]](https://www.zotero.org/google-docs/?8tkae3). Viral genomes were assigned via Hidden Markov Model against the Prokaryotic Virus Orthologous Groups (pVOGs) database using hmmsearch from the HMMER v3.3.2 suite [[16,17]](https://www.zotero.org/google-docs/?npJHvC).

ORFs were predicted using Prodigal v2.6.3 [[18]](https://www.zotero.org/google-docs/?7EC2Fg). Taxonomy was assigned on the basis of a consensus rule, as previously descripted in other works [[19]](https://www.zotero.org/google-docs/?lKpTTg): if at least 20% of the ORFs in a viral genome matched against pVOG models and 60% of those ORFs were assigned to the same taxon, then the viral genome was assigned the corresponding taxonomy. Taxonomy was assigned both to viral MAGs and integrated phages in prokaryotic MAGs.

Functional annotation was carried out on ORFs predicted on prokaryotic and viral genomes using the eggnog-mapper server [[20]](https://www.zotero.org/google-docs/?Fg1Zbg). The completeness of KEGG modules in each microbial genome was calculated with KEMET [[21]](https://www.zotero.org/google-docs/?9qLacD). Furthermore, ORFs annotated with KEGG orthologs belonging to putative alternatives to the Wood-Ljungdahl pathway (WLP) were counted in each MAG to identify potential syntrophic acetate oxidizing *Bacteria* (SAOB). The predicted ORFs were searched against the dbCAN database [[22]](https://www.zotero.org/google-docs/?bjymid) using hmmsearch for finding genes involved in carbohydrate hydrolysis. The MAGs were also analysed with gutSMASH [[23]](https://www.zotero.org/google-docs/?Bao5Rz), in order to find gene clusters related to VFA production and metabolism.

**Detection of induction in integrated prophages**

With the aim of evaluating the induction of putative integrated phages, an analysis was carried out on the eight samples sequenced in this work and extended to 110 samples from the AD database [[24]](https://www.zotero.org/google-docs/?3gbGsY). The aim of the analysis was to check whether prophages detected in the induction experiment were also present (and, possibly, induced) in other, non-related AD communities. MAGs featuring integrated viruses were divided into viral and non viral sequences by manually removing the viral predictions within MAGs and placing them in separate FASTA files. This approach resulted in a data set of 64 prokaryotic MAGs and their 64 respective integrated viral sequences. From each sample, subsamples of 10 million reads were randomly generated and mapped on the 128 data sets. Read coverage of MAG-prophage couples in the different samples was calculated with CheckM coverage. The virus/MAG coverage ratios were calculated and the distributions of their values across samples and across genomes were inspected in order to identify peculiar behaviours. Furthermore, samples were clustered with an average linkage algorithm based on Euclidean distance. Prophages were considered putatively induced in a sample when the log ratio was greater than 10. The distribution of the base 2 logarithm-transformed data has an average of 0.46 and a standard deviation of 2.83. The log2-transformed chosen cutoff is equal to 3.32, which is about one standard deviation above the average.


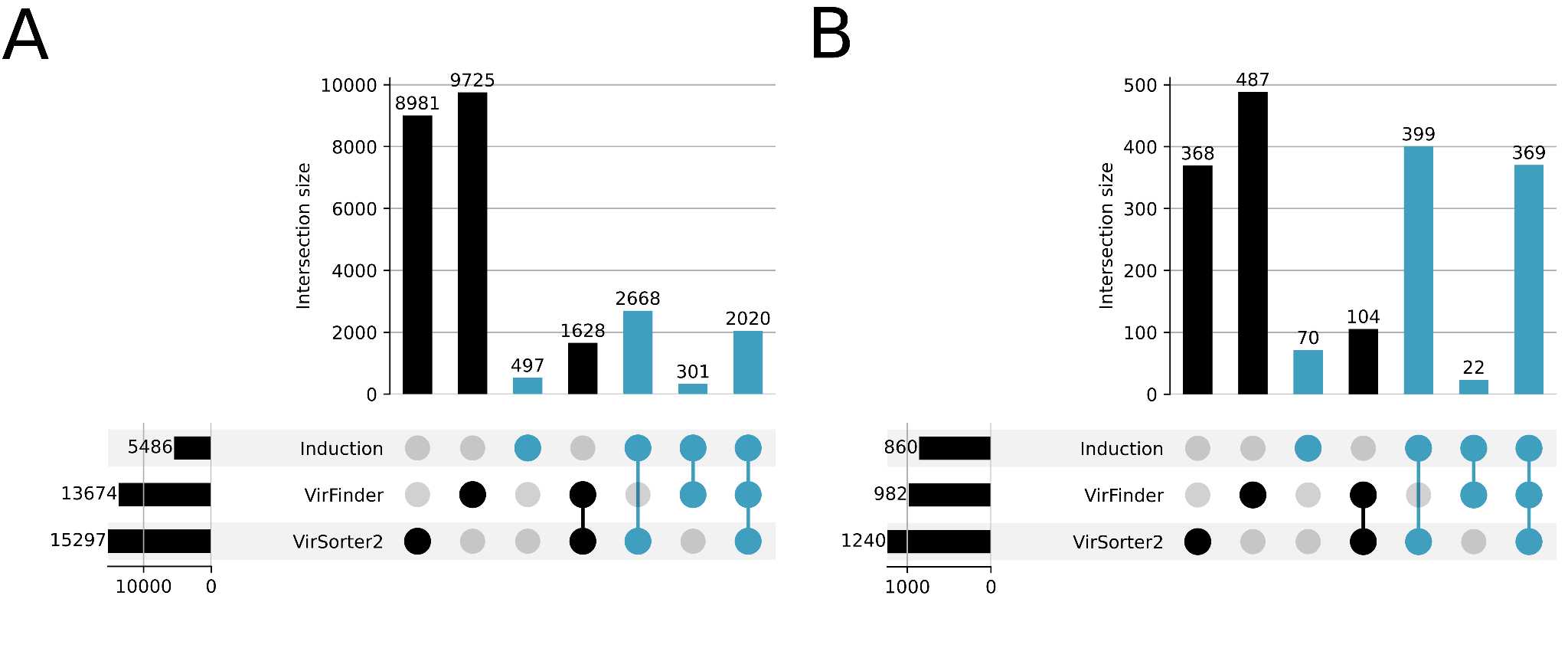


**Additional figure 1** UpSet plot displaying the comparison between the list of viral scaffolds used in the present work (see Methods) and the scaffolds identified by VirSorter2 and VirFinder. The list of viral scaffolds reported in the paper is dubbed “Induction” and the subsets involving it are highlighted in blue. **(A)** Comparison among all viral predictions. **(B)** Comparison among viral predictions performed on scaffolds having length greater than 5000 bp.


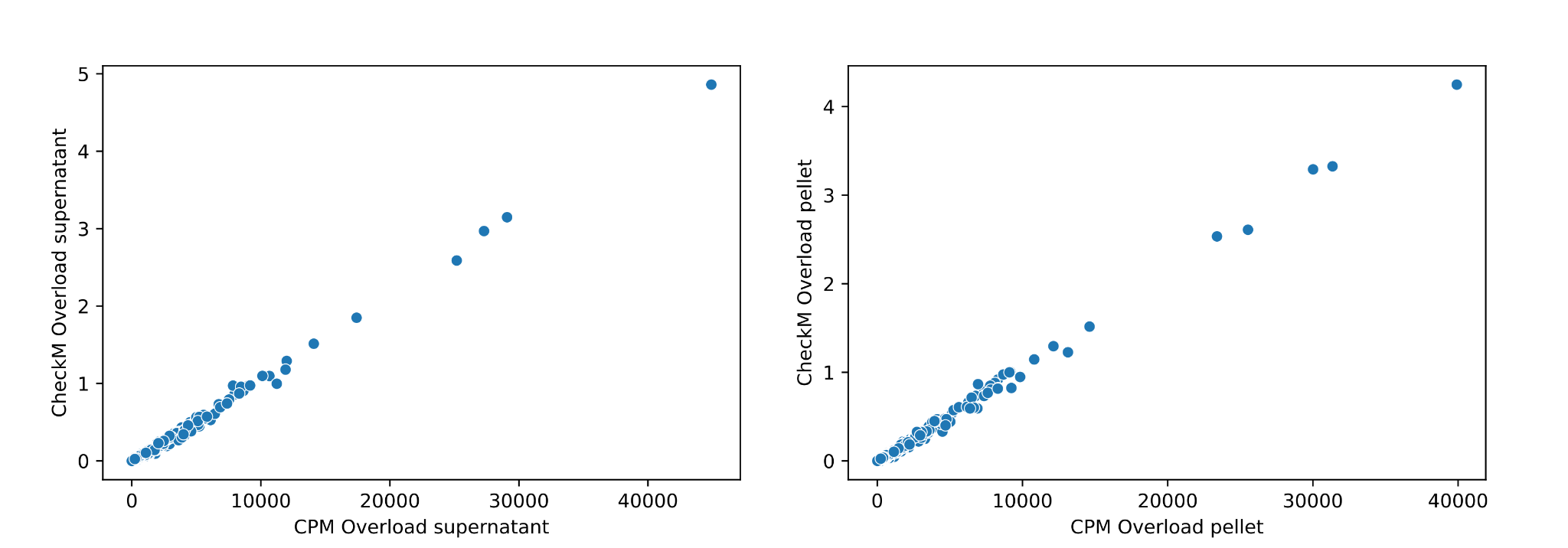

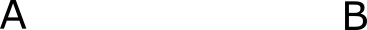

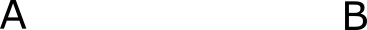


**Additional Figure 2** Scatter plots comparing CPM and CheckM relative abundance values for the organic overload supernatant **(A)** and pellet **(B)** samples.


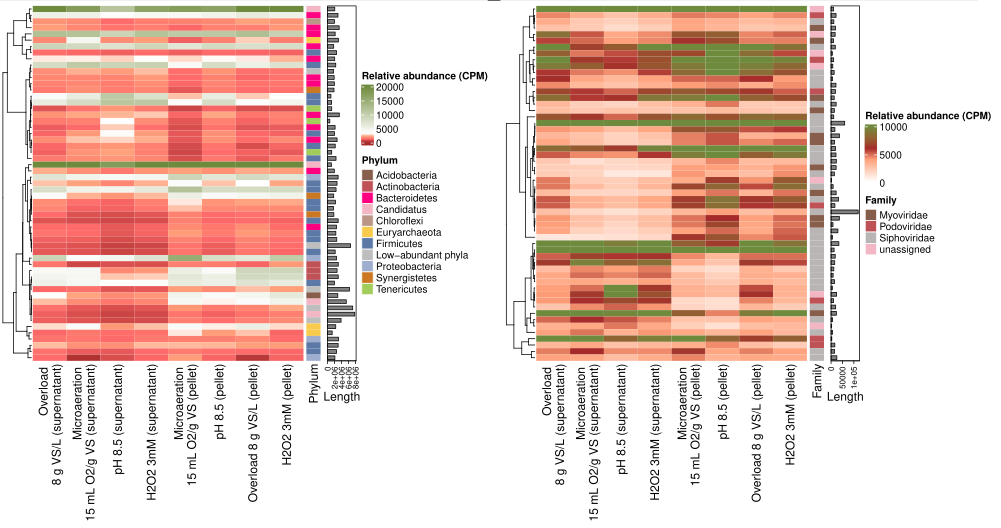


**Additional figure 3** Relative abundance expressed in count per million (CPM) of the most abundant prokaryotic (left) and viral genomes (right). The colored labels on the right of the heatmaps show the genome taxon, i.e. phylum for *Bacteria* and *Archaea*, family for viruses. Genome lengths are reported as bars in the right annotation of the heatmap. The genomes are clustered according to an average linkage algorithm based on Pearson correlation.


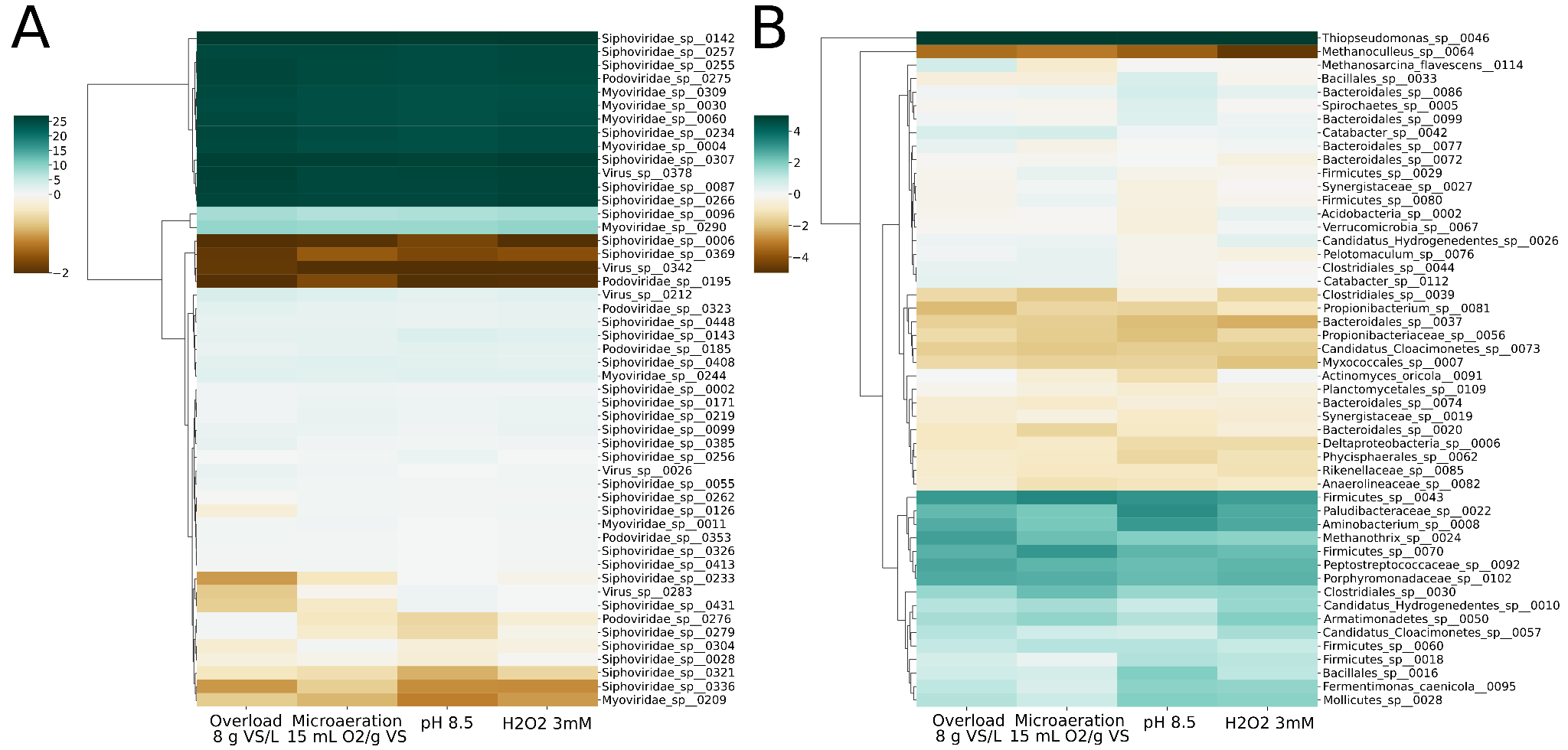


**Additional figure 4** Heatmaps displaying variations of phages and MAGs compared to supernatant and pellet control, respectively. Each column of the heatmap corresponds to a treatment, and the values are the base 2-log ratio between the relative abundance of a genome in the treatment and the relative abundance in the control. A hierarchical clustering tree based on Euclidean distance and performed with average linkage is placed to the left of each heatmap. **(A)** The 50 most abundant phages are displayed. **(B)** The 50 most abundant MAGs are displayed.


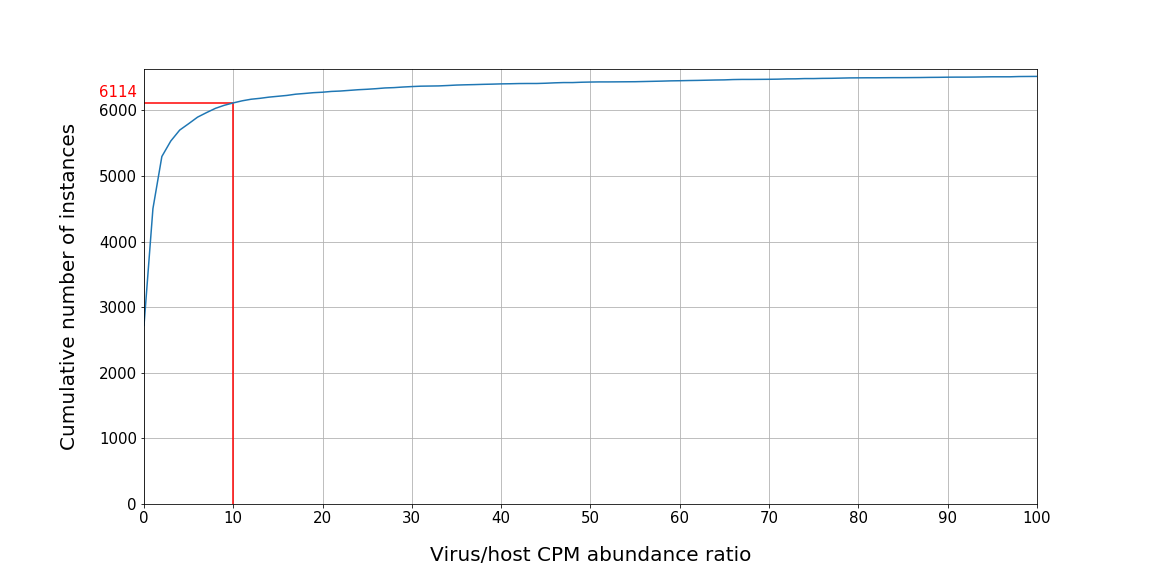


**Additional figure 5** Distribution of the provirus/host CPM abundance ratios across all datasets. The distribution follows an exponential curve, which starts reaching a plateau at around 10. The value has thus been chosen as the cutoff value for determining whether the provirus was putatively induced.

**References**

[1. Bolger AM, Lohse M, Usadel B. Trimmomatic: a flexible trimmer for Illumina sequence data. Bioinformatics. 2014;30:2114–20.](https://www.zotero.org/google-docs/?dyGtix)

[2. Li D, Liu C-M, Luo R, Sadakane K, Lam T-W. MEGAHIT: an ultra-fast single-node solution for large and complex metagenomics assembly via succinct de Bruijn graph. Bioinformatics. 2015;31:1674–6.](https://www.zotero.org/google-docs/?dyGtix)

[3. Gurevich A, Saveliev V, Vyahhi N, Tesler G. QUAST: quality assessment tool for genome assemblies. Bioinformatics. 2013;29:1072–5.](https://www.zotero.org/google-docs/?dyGtix)

[4. Arndt D, Grant JR, Marcu A, Sajed T, Pon A, Liang Y, et al. PHASTER: a better, faster version of the PHAST phage search tool. Nucleic Acids Res. 2016;44:W16–21.](https://www.zotero.org/google-docs/?dyGtix)

[5. Fang Z, Tan J, Wu S, Li M, Xu C, Xie Z, et al. PPR-Meta: a tool for identifying phages and plasmids from metagenomic fragments using deep learning. GigaScience. 2019;8:giz066.](https://www.zotero.org/google-docs/?dyGtix)

[6. Kieft K, Zhou Z, Anantharaman K. VIBRANT: automated recovery, annotation and curation of microbial viruses, and evaluation of viral community function from genomic sequences. Microbiome. 2020;8:90.](https://www.zotero.org/google-docs/?dyGtix)

[7. Nayfach S, Camargo AP, Schulz F, Eloe-Fadrosh E, Roux S, Kyrpides NC. CheckV assesses the quality and completeness of metagenome-assembled viral genomes. Nat Biotechnol. 2021;39:578–85.](https://www.zotero.org/google-docs/?dyGtix)

[8. Ren J, Ahlgren NA, Lu YY, Fuhrman JA, Sun F. VirFinder: a novel k-mer based tool for identifying viral sequences from assembled metagenomic data. Microbiome. 2017;5:69.](https://www.zotero.org/google-docs/?dyGtix)

[9. Guo J, Bolduc B, Zayed AA, Varsani A, Dominguez-Huerta G, Delmont TO, et al. VirSorter2: a multi-classifier, expert-guided approach to detect diverse DNA and RNA viruses. Microbiome. 2021;9:37.](https://www.zotero.org/google-docs/?dyGtix)

[10. Kang DD, Li F, Kirton E, Thomas A, Egan R, An H, et al. MetaBAT 2: an adaptive binning algorithm for robust and efficient genome reconstruction from metagenome assemblies. PeerJ. 2019;7:e7359.](https://www.zotero.org/google-docs/?dyGtix)

[11. Langmead B, Salzberg SL. Fast gapped-read alignment with Bowtie 2. Nat Methods. 2012;9:357–9.](https://www.zotero.org/google-docs/?dyGtix)

[12. Parks DH, Imelfort M, Skennerton CT, Hugenholtz P, Tyson GW. CheckM: assessing the quality of microbial genomes recovered from isolates, single cells, and metagenomes. Genome Res. 2015;25:1043–55.](https://www.zotero.org/google-docs/?dyGtix)

[13. Danecek P, Bonfield JK, Liddle J, Marshall J, Ohan V, Pollard MO, et al. Twelve years of SAMtools and BCFtools. GigaScience. 2021;10:giab008.](https://www.zotero.org/google-docs/?dyGtix)

[14. Virtanen P, Gommers R, Oliphant TE, Haberland M, Reddy T, Cournapeau D, et al. SciPy 1.0: fundamental algorithms for scientific computing in Python. Nat Methods. 2020;17:261–72.](https://www.zotero.org/google-docs/?dyGtix)

[15. Chaumeil P-A, Mussig AJ, Hugenholtz P, Parks DH. GTDB-Tk: a toolkit to classify genomes with the Genome Taxonomy Database. Hancock J, editor. Bioinformatics. 2019;btz848.](https://www.zotero.org/google-docs/?dyGtix)

[16. Eddy SR. Profile hidden Markov models. Bioinformatics. 1998;14:755–63.](https://www.zotero.org/google-docs/?dyGtix)

[17. Grazziotin AL, Koonin EV, Kristensen DM. Prokaryotic Virus Orthologous Groups (pVOGs): a resource for comparative genomics and protein family annotation. Nucleic Acids Res. 2017;45:D491–8.](https://www.zotero.org/google-docs/?dyGtix)

[18. Hyatt D, Chen G-L, LoCascio PF, Land ML, Larimer FW, Hauser LJ. Prodigal: prokaryotic gene recognition and translation initiation site identification. BMC Bioinformatics. 2010;11:119.](https://www.zotero.org/google-docs/?dyGtix)

[19. Camarillo-Guerrero LF, Almeida A, Rangel-Pineros G, Finn RD, Lawley TD. Massive expansion of human gut bacteriophage diversity. Cell. 2021;184:1098-1109.e9.](https://www.zotero.org/google-docs/?dyGtix)

[20. Huerta-Cepas J, Forslund K, Coelho LP, Szklarczyk D, Jensen LJ, von Mering C, et al. Fast Genome-Wide Functional Annotation through Orthology Assignment by eggNOG-Mapper. Mol Biol Evol. 2017;34:2115–22.](https://www.zotero.org/google-docs/?dyGtix)

[21. Palù, M. KEMET [Internet]. University of Padova; 2021. Available from: https://github.com/Matteopaluh/KEMET](https://www.zotero.org/google-docs/?dyGtix)

[22. Yin Y, Mao X, Yang J, Chen X, Mao F, Xu Y. dbCAN: a web resource for automated carbohydrate-active enzyme annotation. Nucleic Acids Res. 2012;40:W445–51.](https://www.zotero.org/google-docs/?dyGtix)

[23. Pascal Andreu V, Roel-Touris J, Dodd D, Fischbach MA, Medema MH. The gutSMASH web server: automated identification of primary metabolic gene clusters from the gut microbiota. Nucleic Acids Res. 2021;49:W263–70.](https://www.zotero.org/google-docs/?dyGtix)

[24. Campanaro S, Treu L, Rodriguez-R LM, Kovalovszki A, Ziels RM, Maus I, et al. New insights from the biogas microbiome by comprehensive genome-resolved metagenomics of nearly 1600 species originating from multiple anaerobic digesters. Biotechnol Biofuels. 2020;13:25.](https://www.zotero.org/google-docs/?dyGtix)
